# Augmenting Mitochondrial Respiration in Immature Smooth Muscle Cells with an *ACTA2* Pathogenic Variant Mitigates Moyamoya-like Cerebrovascular Disease

**DOI:** 10.21203/rs.3.rs-3304679/v1

**Published:** 2023-10-12

**Authors:** Anita Kaw, Ting Wu, Zbigniew Starosolski, Zhen Zhou, Albert J. Pedroza, Suravi Majumder, Xueyan Duan, Kaveeta Kaw, Jose E. E. Pinelo, Michael P. Fischbein, Philip L. Lorenzi, Lin Tan, Sara A. Martinez, Iqbal Mahmud, Laxman Devkota, Heinrich Taegtmeyer, Ketan B. Ghaghada, Sean P. Marrelli, Callie S. Kwartler, Dianna M. Milewicz

**Affiliations:** 1 Division of Medical Genetics, Department of Internal Medicine, McGovern Medical School, The University of Texas Health Science Center at Houston, TX 77030, USA; 2 Department of Neurology, McGovern Medical School, The University of Texas Health Science Center at Houston, McGovern Medical School, 6431 Fannin Street, Houston, TX 77030, USA; 3 Department of Radiology, Baylor College of Medicine, Texas Children’s Hospital, Houston, TX 77030, USA; 4 Department of Cardiothoracic Surgery, Stanford University School of Medicine, Stanford, CA 94305, USA; 5 Metabolomics Core Facility, Department of Bioinformatics & Computational Biology, The University of Texas MD Anderson Cancer Center, Houston, TX, USA; 6 Division of Cardiovascular Medicine, Department of Internal Medicine, McGovern Medical School, The University of Texas Health Science Center at Houston, TX 77030, USA

## Abstract

*ACTA2* pathogenic variants altering arginine 179 cause childhood-onset strokes due to moyamoya disease (MMD)-like occlusion of the distal internal carotid arteries. A smooth muscle cell (SMC)-specific knock-in mouse model (*Acta2*^*SMC-R179C/+*^) inserted the mutation into 67% of aortic SMCs, whereas explanted SMCs were uniformly heterozygous. *Acta2*^*R179C/+*^ SMCs fail to fully differentiate and maintain stem cell-like features, including high glycolytic flux, and increasing oxidative respiration (OXPHOS) with nicotinamide riboside (NR) drives the mutant SMCs to differentiate and decreases migration. *Acta2*^*SMC-R179C/+*^ mice have intraluminal MMD-like occlusive lesions and strokes after carotid artery injury, whereas the similarly treated WT mice have no strokes and patent lumens. Treatment with NR prior to the carotid artery injury attenuates the strokes, MMD-like lumen occlusions, and aberrant vascular remodeling in the *Acta2*^*SMC-R179C/+*^ mice. These data highlight the role of immature SMCs in MMD-associated occlusive disease and demonstrate that altering SMC metabolism to drive quiescence of *Acta2*^*R179C/+*^ SMCs attenuates strokes and aberrant vascular remodeling in the *Acta2*^*SMC-R179C/+*^ mice.

## Introduction

Moyamoya disease (**MMD**), a common cause of pediatric strokes, is characterized by occlusion of the distal internal carotid arteries (**ICAs**) and compensatory collateral vessel formation^1^. The molecular pathogenesis of MMD is poorly understood, but histology of lesions in involved arteries shows lumens filled with neointimal cells that stain positive for smooth muscle cell (**SMC**) markers and lack atherosclerotic features, such as macrophage foam cells and cholesterol and calcium deposition^2,3^. Genetic variants are major risk factors for MMD^4–6^. Heterozygous pathogenic variants in *ACTA2* predispose to thoracic aortic disease, and a subset of these variants also predispose to MMD-like disease^7^. Cerebrovascular disease in these individuals is characterized by typical occlusive lesions in the distal ICAs, but also unique features, such as a lack of collateral vessels, dilatation of proximal ICA, straightened cerebral arteries, and periventricular white matter hyperintensities^8–10^. *De novo ACTA2* variants disrupting arginine 179 to any of six amino acids lead to Smooth Muscle Dysfunction Syndrome (**SMDS**), a childhood-onset condition characterized by the earliest onset of MMD-like cerebrovascular disease and thoracic aortic disease, along with patent ductus arteriosus, pulmonary hypertension, aberrant lung development, fixed dilated pupils, gut malrotation, hypoperistalsis, and hypotonic bladder^8,11,12^. Similar to MMD lesions, involved arteries in SMDS patients have neointimal lesions filled with SMC marker-positive cells, along with thickened arterial walls due to increased area of the medial layer^13^.

*ACTA2* encodes the most abundant protein in vascular SMCs, the SMC-specific isoform of α-actin (**SMA**)^14–16^. Monomers of SMA polymerize to form the thin filaments of SMC contractile units, and *ACTA2* R179H disrupts SMA polymerization^17^. We engineered a mouse model with an SMC-specific constitutive insertion of Arg179Cys mutation into an *Acta2* allele, termed *Acta2*^*SMC-R179C/+*^ mice^18^. Single cell RNA sequencing (**scRNA-seq**) of ascending aortic tissue from these mice revealed that 67% of the SMCs have correct insertion of the mutation, which leads to two phenotypically distinct SMC clusters, wildtype (**WT**) SMCs and SMCs heterozygous for the *Acta2* R179C mutation^18^. The mutant SMCs are incompletely differentiated based on decreased expression of SMC-specific markers (e.g., *Myh11*, *Actg2*, *Tagln*, and *Cnn1*), and *in vitro* ATAC-seq identified increased expression and accessibility of *Klf4*, a transcription factor associated with SMC plasticity^19,20^. Explanted SMCs from *Acta2*^*SMC-R179C/+*^ ascending aortas are uniformly heterozygous for the *Acta2* R179C mutation based on RNA sequencing and show reduced differentiation, continued expression of pluripotency markers, and increased proliferation and migration (designated as *Acta2*^*R179C/+*^ SMCs)^18,20^. We went on to identify a novel role of SMA in the nucleus that is critical for SMC differentiation, and mutant SMA with an altered R179 disrupts this nuclear function. Thus, we propose that the loss of functional nuclear SMA underlies the failure of *Acta2*^*R179C/+*^ SMCs to properly differentiate into contractile and quiescent SMCs^20^.

Undifferentiated stem cells rely more heavily on glycolysis rather than oxidative phosphorylation (**OXPHOS**) for production of ATP and nucleotides^21^. Stem cells have immature mitochondria with poorly developed mitochondrial infrastructure and lower membrane potential associated with reduced electron transport chain (**ETC**) function^21–23^. With differentiation, cells transition to more efficient ATP production through the mitochondrial ETC and OXPHOS^23–25^. Moreover, altering stem cell metabolism from glycolysis to OXPHOS can drive cellular differentiation, and this metabolic shift is required for complete cellular differentiation and quiescence^26–29^. Here, we show that lack of differentiation of *Acta2*^*R179C/+*^ SMCs is associated with high glycolytic flux, and exposure to nicotinamide riboside (NR) not only decreases glycolysis and increases OXPHOS, but also drives differentiation and decreases migration. Furthermore, we demonstrate that *Acta2*^*SMC-R179C/+*^ mice have strokes and intraluminal MMD-like occlusive lesions after carotid artery injury, whereas the WT mice have no strokes and patent lumens. Treatment with NR prior to carotid artery injury attenuates strokes, MMD-like lumen occlusions, and aberrant vascular remodeling in the *Acta2*^*SMC-R179C/+*^ mice.

## Results

### Acta2^R179C/+^ SMCs have increased glycolytic flux and augmenting OXPHOS drives differentiation and quiescence of mutant cells

**Figure 1.**
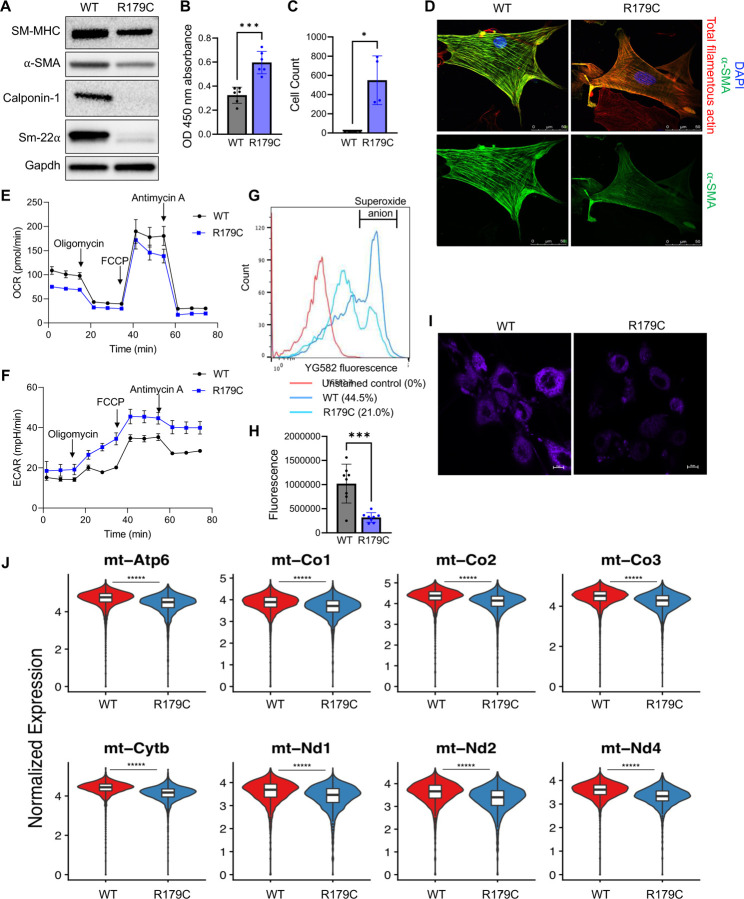
*Acta2*^*R179C/+*^ SMCs Exhibit Stem Cell-Like Properties Including Reduced OXPHOS. **A.** Immunoblot analysis shows reduced levels of SMC contractile proteins in *Acta2*^*R179C/+*^ SMCs compared to WT. **B.** *Acta2*^*R179C/+*^ SMCs proliferate faster than WT SMCs based on BrdU ELISA. **C.** Transwell migration assay shows that *Acta2*^*R179C/+*^ SMCs migrate faster than WT SMCs. **D.** *Acta2*^*R179C/+*^ SMCs have reduced αSMA filament formation compared to WT. **E.** *Acta2*^*R179C/+*^ SMCs have reduced basal, ATP-linked, and maximal OCR compared to WT. **F.** *Acta2*^*R179C/+*^ SMCs have increased ECAR, indicating increased proton generation primarily produced by lactate formation during anaerobic glycolysis. **G.** *Acta2*^*R179C/+*^ SMCs generate decreased mitochondrial superoxide compared to WT. **H-I.** Live cell confocal microscopy at 100X magnification shows that *Acta2*^*R179C/+*^ SMCs have reduced MTDR fluorescence intensity compared to WT. **J.** *Acta2*^*SMC-R179C/+*^ aortic SMCs show reduced expression of mtDNA-encoded electron transport chain complex subunits *mt-Atp6*, *mt-Co1*, *mt-Co2*, *mt-Co3*, *mt-Cytb*, *mt-Nd1*, *mt-Nd2*, and *mt-Nd4*. OXPHOS; oxidative phosphorylation. SMC; smooth muscle cell. WT; wildtype. OCR; oxygen consumption rate. ECAR, extracellular acidification rate. MTDR; MitoTracker Deep Red. mtDNA; mitochondrial DNA. *p<0.05, **p<0.01, ***p<0.001, ****p<0.0001, *****p<1×10^−100^.

We confirmed *Acta2*^*R179C/+*^ SMCs explanted from the ascending aorta have reduced levels of SMC differentiation markers (smooth muscle myosin heavy chain [**SM-MHC**], SMA, transgelin [**SM-22α**], and calponin-1) and increased migration and proliferation, along with decreased SMA filament formation (Fig. 1A–D, S1A)^17^. Seahorse assays identified that *Acta2*^*R179C/+*^ SMCs maintain additional stem cell traits, including increased extracellular acidification rate (**ECAR**, indicative of glycolysis) and lower oxygen consumption rate (**OCR**, reflecting ETC activity) compared to WT SMCs (Fig. 1E–F). Consistent with decreased ETC function, *Acta2*^*R179C/+*^ SMCs also generate less mitochondrial reactive oxygen species (**ROS**) (Fig. 1G). MitoTracker Deep Red (**MTDR**) staining was lower in *Acta2*^*R179C/+*^ SMCs compared to WT SMCs, indicating reduction in mitochondrial function or mass (Fig. 1H–I). Additionally, scRNA-seq data from the *Acta2*^*SMC-R179C/+*^ mouse aortas indicate that the mutant SMC cluster has significantly reduced expression of 8 of 13 mitochondrial DNA (**mtDNA**)-encoded ETC complex subunits (*mt-Atp6*, *mt-Co1*, *mt-Co2*, *mt-Co3*, *mt-Cytb*, *mt-Nd1*, *mt-Nd2*, *mt*-*Nd4*) compared to the WT SMC cluster (Fig. 1J)^18^.

**Figure 2.**
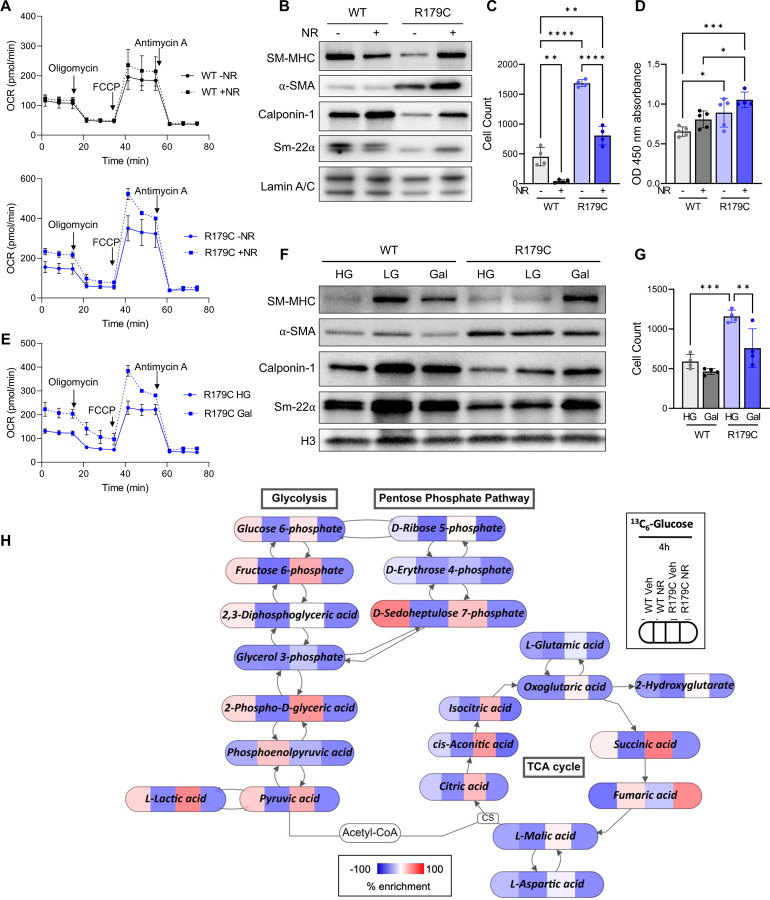
NR Induces Differentiation and Quiescence in *Acta2*^*R179C/+*^ SMCs. **A.** WT and *Acta2*^*R179C/+*^ SMCs increase ATP-linked and maximal OCR in response to NR. **B.** *Acta2*^*R179C/+*^ SMCs increase levels of contractile proteins in response to NR stimulation. **C.** Both WT and *Acta2*^*R179C/+*^ SMCs reduce migration in response to NR stimulation. **D.** Proliferation is unchanged in WT and *Acta2*^*R179C/+*^ SMCs in response to NR exposure. **E.** *Acta2*^*R179C/+*^ SMCs increase ATP-linked and maximal OCR in response to galactose. **F.** WT SMCs increase differentiation markers when cultured in both low-glucose and galactose while *Acta2*^*R179C/+*^ SMCs only increase differentiation markers when cultured in galactose. **G.** Replacing high glucose media with galactose-containing media reduces migration in *Acta2*^*R179C/+*^ SMCs. **H.** *Acta2*^*R179C/+*^ SMCs have increased glycolytic and TCA flux compared to WT, and NR reduces glycolytic, TCA, and PPP flux in both WT and *Acta2*^*R179C/+*^ SMCs. NR; nicotinamide riboside. SMC; smooth muscle cell. WT; wildtype. OCR; oxygen consumption rate. PPP; pentose phosphate pathway. *p<0.05, **p<0.01, ***p<0.001, ****p<0.0001.

Since driving oxidative metabolism can differentiate stem cells, WT and *Acta2*^*R179C/+*^ SMCs were exposed to NR, a NAD+ precursor, which significantly boosts OXPHOS based on increases in basal, ATP-linked, and maximal OCR in the mutant SMCs (Fig. 2A). NR treatment also increases SMC differentiation markers in the *Acta2*^*R179C/+*^ SMCs (Fig. 2B, S1B) and decreases migration of the *Acta2*^*R179C/+*^ SMCs (Fig. 2C). NR treatment does not alter proliferation (Fig. 2D) or apoptosis or necrosis in *Acta2*^*R179C/+*^ or WT SMCs (Fig. S2A–B). *Acta2*^*R179C/+*^ SMCs were also exposed to media with galactose without glucose, along with low- and high-glucose media. Since galactose does not produce ATP during glycolysis, cells boost OXPHOS using other sources of energy^30–32^. Galactose-containing media also increases OCR in *Acta2*^*R179C/+*^ SMCs compared to high glucose media (Fig. 2E). While WT SMCs exhibit increased differentiation in low glucose media as expected^30–32^, *Acta2*^*R179C/+*^ SMCs only increase levels of differentiation markers and decrease migration in galactose-containing media (Fig. 2F–G, S1C).

Metabolomics experiments with carbon tracers were used to assess flux through glycolysis, the pentose phosphate pathway (PPP), and the Krebs cycle in *Acta2*^*R179C/+*^ SMCs in the presence and absence of NR. When compared to WT SMCs, *Acta2*^*R179C/+*^ exhibit increased flux of 1,2-^13^C_2_-glucose through glycolysis based on increased fructose 6-phosphate through lactic acid levels without significant differences in flux through PPP, along with increased levels of Krebs cycle metabolites (L-glutamic acid, oxoglutaric acid, 2-hydroxyglutarate-succinic acid, furmaric acid, L-malic acid, L-aspartic acid, citric acid, cis-aconitic acid, and isocitric acid) (Fig. 2H). NR treatment markedly decreases glucose flux through the glycolysis, the PPP, and the Krebs cycle in both the WT and mutant SMCs (Fig. 2H).

### Electric transport chain defects in the Acta2^R179C/+^ SMCs are reversed by NR

**Figure 3.**
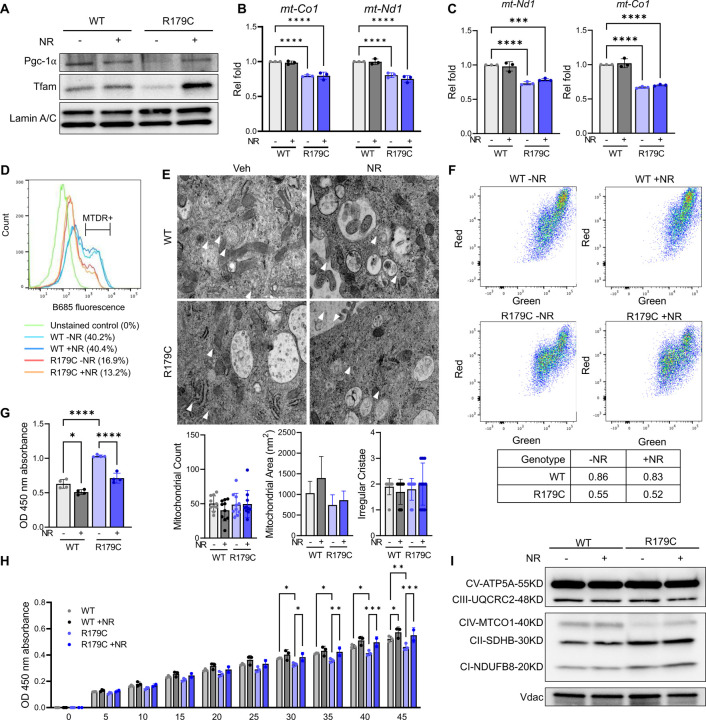
*Acta2*^*R179C/+*^ SMCs Exhibit Mitochondrial Dysfunction that is Restored by NR. **A.** *Acta2*^*R179C/+*^ SMCs have reduced levels of mitochondrial markers Pgc-1α and Tfam compared to WT, which increase with NR stimulation. **B.** *Acta2*^*R179C/+*^ SMCs have decreased mtDNA levels compared to WT, which are unaffected by NR treatment. **C.** Transcript levels of *mt-Co1* and *mt-Nd1* remain are reduced in *Acta2*^*R179C/+*^ SMCs even with NR treatment. **D.** MTDR staining is unchanged with NR stimulation of WT and *Acta2*^*R179C/+*^ SMCs. **E.** Vehicle- and NR-treated WT and *Acta2*^*R179C/+*^ SMCs have similar mitochondrial counts, area, and irregular cristae. **F.** *Acta2*^*R179C/+*^ SMCs have reduced JC-1 staining intensity compared to WT, which is not altered with NR stimulation. **G.** *Acta2*^*R179C/+*^ SMCs produce more lactate compared to WT, which decreases with NR treatment. **H.** *Acta2*^*R179C/+*^ SMCs have reduced complex I activity compared to WT, which increases with NR stimulation. **I.** *Acta2*^*R179C/+*^ SMCs have lower levels of mt-Co1 protein, which increases with NR treatment. SMC; smooth muscle cell. NR; nicotinamide riboside. WT; wildtype. mtDNA; mitochondrial DNA. MTDR; MitoTracker Deep Red. *p<0.05, **p<0.01, ***p<0.001, ****p<0.0001.

Peroxisome proliferator-activated receptor gamma coactivator 1-alpha (**PGC-1α**) promotes mitochondrial biogenesis through increased transcription of mitochondrial transcription factor A (**TFAM**), which subsequently increases mtDNA replication, ETC gene expression, and OXPHOS^33^. PGC-1α and TFAM levels are reduced in *Acta2*^*R179C/+*^ SMCs, and both increase with NR treatment (Fig. 3A, S3A). Mitochondrial DNA levels are also decreased in *Acta2*^*R179C/+*^ SMCs compared to WT SMCs, but do not increase with NR treatment (Fig. 3B). Furthermore, NR treatment does not increase the low expression of mtDNA-encoded ETC complex subunits, *mt-Co1* and *mt-Nd1* (Fig. 3C), or the low MTDR staining in *Acta2*^*R179C/+*^ SMCs (Fig. 3D). Despite these findings, transmission electron microscopy (**TEM**) analyses found no differences in mitochondrial cristae morphology, count, or area based on genotype or NR treatment among these SMCs (Fig. 3E). Stem cell mitochondria have a reduced mitochondrial membrane potential^34^. JC-1 is a mitochondrial potential-dependent stain which exhibits green fluorescence in the monomeric form, and upon accumulation and aggregation, JC-1 emits red fluorescence^35^. *Acta2*^*R179C/+*^ SMCs display reduced potential compared to WT SMCs based on JC-1 staining, which shows reduced red:green fluorescence consistent with depolarization, and does not change with NR treatment (Fig. 3F). These data suggest that the reduced oxidative capacity in *Acta2*^*R179C/+*^ SMCs is due to mitochondrial dysfunction rather than reduced mitochondrial mass.

Lactate assays indicate increased anaerobic glycolytic activity in *Acta2*^*R179C/+*^ SMCs compared to WT and that NR reduces anaerobic glycolysis in *Acta2*^*R179C/+*^ SMCs (Fig. 3G). Assessment of the individual ETC complexes identifies that *Acta2*^*R179C/+*^ SMCs have reduced complex I activity and decreased protein levels of complex IV subunit mt-Co1, and both are restored with NR treatment (Fig. 3H–I, S3B). Single cell transcriptomic data also shows that the mutant SMC cluster has decreased expression of *mt-Co1* (Fig. 1J). Despite increased ETC activity with NR treatment, *Acta2*^*R179C/+*^ SMCs, ROS levels do not increase with NR treatment (Fig. S2C). Therefore, NR reduces glycolytic metabolism and improves mitochondrial function in *Acta2*^*R179C/+*^ SMCs by increasing ETC activity and not by augmenting mitochondrial biogenesis.

### Acta2^SMC-R179C/+^ mice have MMD-like occlusive lesions with vascular injury that are prevented with NR treatment

**Figure 4.**
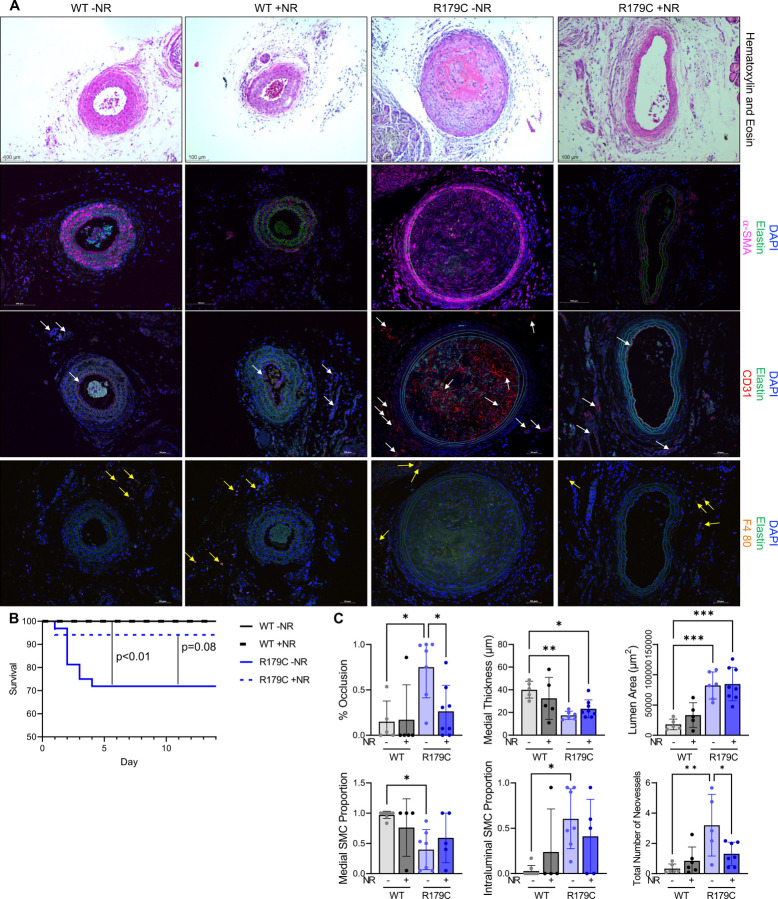
NR Reduces Intraluminal Lesion Burden and Enhanced Neovascularization in LCAL-Injured *Acta2*^*SMC-R179C/+*^ Mice. **A, C.** Histology shows unresolved neointima-thrombi proximal to ligation site in *Acta2*^*SMC-R179C/+*^ mice 21 days post LCAL (compared to patent left carotid arteries in WT mice) that resolve with NR treatment. *Acta2*^*SMC-R179C/+*^ arteries have thinned medial layers and increased lumen area, neither of which are reversed with NR treatment, and increased intraluminal SMA+ cells, which is partially reversed with NR. *Acta2*^*SMC-R179C/+*^ arteries also have increased CD31+ neovessels (white arrow) surrounding the left carotid artery as well as endothelial infiltration into the neointima (white arrow) compared to WT and reduced number of neovessels in NR-treated compared to untreated *Acta2*^*SMC-R179C/+*^ mice 21 days post LCAL. F4/80 staining shows little to no macrophage infiltration (yellow arrow) in the occlusive lesion or within the adventitia in all groups. **B.** *Acta2*^*SMC-R179C/+*^ mice that undergo LCAL have reduced survival compared to WT mice, and NR treatment partially improves survival of *Acta2*^*SMC-R179C/+*^ mice. Survival cohort includes mice that were sacrificed at days 14 and 21 for tissue collection. All mice that survived past day 14 survived to day 21. LCAL; left carotid artery ligation. WT; wildtype. NR; nicotinamide riboside. *p<0.05, **p<0.01, ***p<0.001, ****p<0.0001.

Based on Microfil perfusion and *ex vivo* computed tomography (**CT**) imaging, the *Acta2*^*SMC-R179C/+*^ mice have significant straightening of the left middle cerebral artery (**MCA**) based on reduced tortuosity (p<0.01) and stenosis of the basilar artery based on diameter (p<0.05) (Fig. S4A–B). Since the *Acta2*^*SMC-R179C/+*^ mice do not have overt evidence of decreased cerebral blood flow, left carotid artery ligation (**LCAL**) was performed in *Acta2*^*SMC-R179C/+*^ and WT mice at 8 weeks of age, and the mice were assessed at 21 days post-ligation. LCAL induces clot formation in the injured artery, which is followed by the recruitment of hematopoietic cells and medial SMCs, resolution of the clot, and patency of the lumen by 21 days^36^. After LCAL, 28% of *Acta2*^*SMC-R179C/+*^ mice died within 5 days while none of the WT mice died (n=9/32 vs. n=0/28; p<0.01) (Fig. 4B). Three of *Acta2*^*SMC-R179C/+*^ mice had behavioral changes consistent with a left-sided stroke, including contralateral sensorimotor and strength deficits, circling, and loss of balance. Histological staining of the brain from one of these mice shows a large ischemic infarct in the left cerebral hemisphere (Fig. S9A). Necropsy of mutant mice did not show hemorrhagic strokes, aortic ruptures or intramural hematomas, or gut/bladder distensions.

To characterize pathology throughout the injured artery, the left carotid arteries were transversely sectioned 21 days after LCAL from the ligation site to the proximal origin of the artery, and a section was assessed every 75 μm (Fig. S5). WT mice have patent lumens throughout the injured arteries, with little to no neointimal lesions or medial thickening (Fig. 4A, 4C, S5, S7A–C). In contrast, surviving *Acta2*^*SMC-R179C/+*^ mice have enlarged left carotid arteries with thinned medial layers and flattened elastic fibers, and sections near the ligation site showed lumens filled with cells and matrix material (Fig. 4A, 4C, S5, S7A–C). Immunostaining identified SMA+ cells present in the neointimal lesions and medial layers of the LCAL-injured *Acta2*^*SMC-R179C/+*^ mice but only in the medial layer of the WT arteries (Fig. 4A, 4C). CD31 immunostaining for endothelial cells identified robust staining in the intima and adventitia layers of the *Acta2*^*SMC-R179C/+*^ carotids, consistent with neovascularization, whereas WT carotids showed little or no staining (p<0.01, neovessel quantification) (Fig. 4A, 4C, S8B). Immunostaining for the F4/80 macrophage marker identified few scattered macrophages in the adventitia surrounding the left carotid arteries of WT and *Acta2*^*SMC-R179C/+*^ mice but no staining in the intraluminal lesions (Fig. 4A).

To determine if boosting OXPHOS to drive quiescence of the mutant SMCs attenuates MMD-like occlusive lesion formation in LCAL-injured *Acta2*^*SMC-R179C/+*^ mice, *Acta2*^*SMC-R179C/+*^ and WT mice were treated with vehicle or 1000 mg/kg NR every other day beginning five days prior to LCAL and continuing until 21 days post-LCAL. NR treatment shows a trend of preventing ischemic strokes deaths in *Acta2*^*SMC-R179C/+*^ mice with LCAL (n=9/32 untreated versus 1/17 NR-treated mutant mice; p=0.08) (Fig. 4B). With NR treatment, *Acta2*^*SMC-R179C/+*^ injured arteries remain enlarged, but neointimal lesions and adventitial neovascularization are significantly reduced compared to untreated *Acta2*^*SMC-R179C/+*^ mice (Fig. 4A, 4C, S5, S7A–C). No differences in the right carotid arteries or diameter of the root and ascending aorta based on genotype or treatment were identified (Fig. S6, S7D–F, S8A).

**Figure 5.**
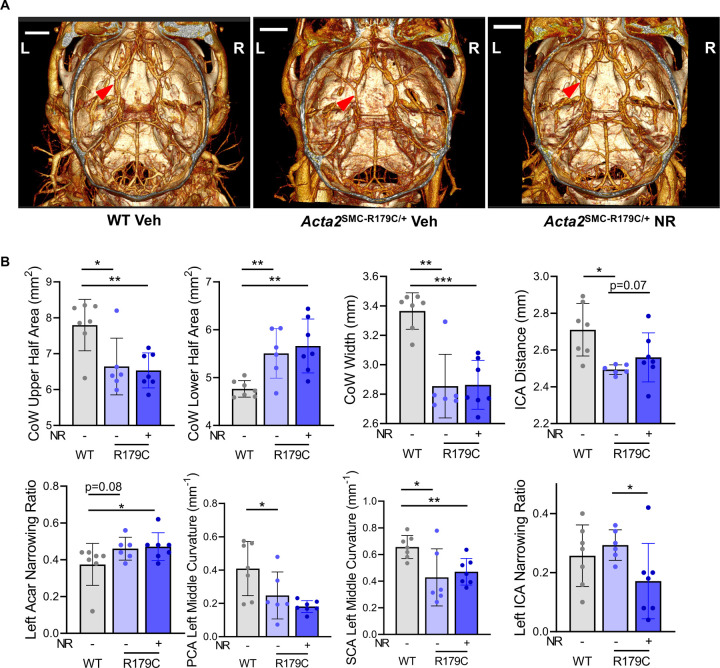
NR Attenuates Post-Occlusion Stenosis of the Large Cerebral Arteries in LCAL-Injured *Acta2*^*SMC-R179C/+*^ Mice. **A-B.** *In vivo* nanoparticle contrast-enhanced CT imaging shows trend of reduced CoW area, CoW width, ICA distance, left-sided CoW arterial diameter (demonstrated by left Acar and ICA narrowing ratio), and left-sided CoW arterial straightening (demonstrated by left PCA and SCA middle curvature) in *Acta2*^*SMC-R179C/+*^ mice compared to WT and a trend of increased CoW arterial diameter in NR-treated compared to untreated *Acta2*^*SMC-R179C/+*^ mice 21 days post-LCAL. NR; nicotinamide riboside. LCAL; left carotid artery ligation. CT; computed tomography. CoW; Circle of Willis. ICA; internal carotid artery. Acar; anterior cerebral and communicating arteries. PCA; posterior cerebral artery. SCA; superior cerebellar artery. WT; wildtype. *p<0.05, **p<0.01, ***p<0.001, ****p<0.0001.

*In vivo* nanoparticle contrast enhanced CT imaging of the brain with a long-circulating liposomal-iodine blood pool contrast agent was used to further assess cerebrovascular changes in *Acta2*^*SMC-R179C/+*^ mice 21 days post-LCAL^37^. Post LCAL, *Acta2*^*SMC-R179C/+*^ mice have a narrowed Circle of Willis (**CoW**) with changes in CoW area (reduced upper half and increased lower half), reduced CoW width (MCA-MCA), and reduced distance between ICAs (Fig. 5A–B). The mutant mice have significant straightening (posterior cerebral artery [**PCA**] and superior cerebellar artery [**SCA**], p=0.05) compared to WT and trend towards left-sided arterial narrowing (anterior cerebral and communicating arteries [**Acar**], p=0.08) (Fig. 5A–B). NR treatment increases the left ICA diameter in the *Acta2*^*SMC-R179C/+*^ mice (p<0.05) and shows a trend to improve the distance between the ICAs (p=0.07) (Fig. 5A–B). Histologic analyses of the left and right ICAs show no neointimal formation or differences in medial thickness post-LCAL, but there was a trend of reduced arterial circumference in the left internal carotid arteries of mutant mice which increases with NR treatment (Fig. S9B–D). Staining for activated microgliosis (Iba1) shows no differences among the mouse brains (Fig. S10). Reactive astrocytosis staining (GFAP) found that the NR-treated WT mouse brains show significantly decreased ipsilateral reactive astrocytosis staining compared to vehicle and NR-treated *Acta2*^*SMC-R179C/+*^ brains and significantly reduced contralateral reactive astrocytosis compared to all other groups (Fig. S10).

**Figure 6.**
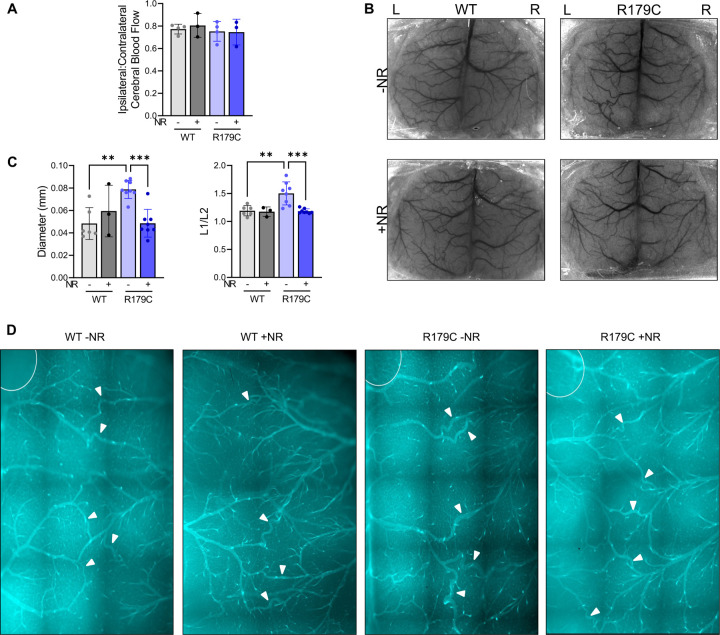
*Acta2*^*SMC-R179C/+*^ Mice Treated with NR Maintain Cerebral Perfusion Post-LCAL Despite Reduced Leptomeningeal Collateral Remodeling Likely Through Increased CoW Circulation. **A.** Surviving vehicle- and NR-treated WT and *Acta2*^*SMC-R179C/+*^ mice show consistently reduced ipsilateral:contralateral CBF 21 days post LCAL. **B.** Sample LSCI images of untreated and NR-treated WT and *Acta2*^*SMC-R179C/+*^ mice 21 days post LCAL. **C.** Vehicle-treated *Acta2*^*SMC-R179C/+*^ mice exhibit increased leptomeningeal collateral tortuosity and diameter compared to WT, and NR attenuates this phenotype. **D.** *Acta2*^*SMC-R179C/+*^mice have increased diameter and tortuosity of lectin-stained collateral vessels and NR attenuates this phenotype. LSCI; laser speckle contrast imaging. LCAL; left carotid artery ligation. WT; wildtype. NR; nicotinamide riboside. CBF; cerebral blood flow. *p<0.05, **p<0.01, ***p<0.001, ****p<0.0001.

Lastly, cerebral blood flow (**CBF**) in the microcirculation assessed using laser speckle contrast imaging (**LSCI**) shows a consistent reduction of ipsilateral CBF of 20–25% compared to contralateral CBF with LCAL in all mice (Fig. 6A–B, S11). Induced ischemic strokes in mice identified two collateral systems that provide bidirectional blood flow between cerebral hemispheres and arterial territories, the CoW and the distalmost branches of the leptomeningeal arteries^38^. LCAL is predicted to trigger compensatory blood flow from the right ICA and posterior circulation toward cerebral arteries ipsilateral to the LCAL through these two collateral systems. Assessment of the leptomeningeal collateral remodeling in vehicle- and NR-treated WT and *Acta2*^*SMC-R179C/+*^ mouse brains 21 days after LCAL reveals increased left-sided inner leptomeningeal collateral diameter and tortuosity in the anterior cerebral arterial and middle cerebral arterial territories of *Acta2*^*SMC-R179C/+*^ brains post-LCAL compared to WT, and NR treatment significantly reduces this remodeling to levels similar to that in the WT brains (Fig. 6C–D, S12).

## Discussion

Patients with SMDS due to pathogenic variants altering SMA arginine 179 have MMD-like cerebrovascular disease, with ischemic lesions evident shortly after birth and up to 40% of the patients having critical stenoses of the ICA and/or its branches^10^. The molecular pathogenesis of MMD in these patients, and MMD overall, is poorly understood. Our data indicate that *ACTA2* p.Arg179 variants disrupt differentiation of neural crest stem cells (**NCCs**) to SMCs, leading to decreased levels of differentiation markers, continued expression of pluripotency markers, and increased migration and proliferation in the mutant SMCs^20^. The cerebrovascular arteries in patients with *ACTA2* p.Arg179 variants are confirmed to have neointimal lesions filled with SMA+ cells, and the injured carotid arteries in the *Acta2*^*SMC-R179C/+*^ mice are similarly filled with SMA+ staining cells, whereas the arteries in WT mice have patent lumens^9,13^. The lack of complete differentiation and quiescence of the *Acta2*^*R179C/+*^ SMCs suggests the mutant SMCs excessively migrate from the arterial wall into the lumen with clot formation with arterial injury. In addition, the mutant SMCs fail to resolve the clot and instead proliferate to contribute to the formation of occlusive lesions. Given the selective expression of *Acta2* in SMCs, the data presented here emphasize the role of an altered SMC phenotype, characterized by incomplete differentiation and quiescence, in the molecular pathogenesis of MMD occlusive lesions.

Seahorse analyses, metabolomics, and other *in vitro* assays indicate that *Acta2*^*R179C/+*^ SMCs retain another feature of stem cells: increased glycolytic flux to generate energy rather than switching to oxidative metabolism. As previously described for stem cells, we also found that decreasing glycolysis and increasing OXPHOS with NR treatment drives differentiation and decreases migration of *Acta2*^*R179C/+*^ SMCs. Unexpectedly, the increased OXPHOS with NR exposure was driven through increased levels and function of specific proteins in the ETC, not by increasing mitochondrial biogenesis. NR treatment in *Acta2*^*SMC-R179C/+*^ mice significantly attenuates occlusive lesion formation in injured carotid arteries, suggesting that NR-induced quiescence of the SMCs prevents excessive mutant SMC migration into the lumen with clot formation. The fact that NR decreases migration but not proliferation of *Acta2*^*R179C/+*^ SMCs implicates excessive migration into the lumen with vascular injury as a critical step in aberrant remodeling leading to occlusive lesions. Our data also suggests that NR treatment may prevent deaths due to strokes and significantly reverses remodeling of the leptomeningeal collaterals with LCAL in *Acta2*^*SMC-R179C/+*^ mice, both indicative of improved cerebral blood flow. Thus, these results define a SMC phenotype of disrupted differentiation and increased migration as the underlying cellular alteration contributing to MMD occlusive lesions in SMDS patients and identifies potential therapies targeting SMC metabolism to prevent these lesions.

It is notable that the MMD-like lesions are elicited in *Acta2*^*SMC-R179C/+*^ mice despite the mice being mosaic for the mutation. SMCs in atherosclerotic plaques arise from the migration and clonal expansion of a few medial SMCs^39^. Thus, the occluded arteries in individuals with MMD and in our mouse model may be derived from just a few medial SMCs primed to migrate into the lumen and then proliferate. These observations have implications for gene therapy that use genome editing technology to correct the *ACTA2* p.Arg179 variants in SMCs: this approach may not prevent MMD cerebrovascular occlusive lesions unless most, if not, all, the hypermigratory SMCs are edited to correct the mutation.

The maturation of the mitochondrial infrastructure, through enrichment of cristae, elongation, and increased mass, increases oxidative metabolic capacity and occurs with the differentiation of stem cells into specialized cell types^26,40^. Likewise, the activity of ETC complexes, including complex I, is essential for the differentiation of stem cells, and disrupted complex function is associated with impaired stem cell differentiation^27,29^. SMCs in the ascending aorta and cerebrovascular circulation are derived from NCCs, and NCCs are reliant on glycolytic flux for delamination and migration during embryonic development^41–43^. NCC differentiation to quiescent, non-migratory cells is accompanied by a transition to OXPHOS, and this transition is critical for NCC differentiation into different cell types^42^. The altered phenotype of the *Acta2*^*R179C/+*^ SMCs is defined by a failure to differentiate, which is associated with significantly increased migration, similar to the NCCs. Although NR only partially rescues differentiation, it effectively decreases the migration of *Acta2*^*R179C/+*^ SMCs to the level of WT SMCs, further emphasizing critical importance of migration in lesion pathogenesis.

Although NR decreases glycolysis and increases OXPHOS in the *Acta2*^*R179C/+*^ SMCs, it does so in an unexpected manner. The NAD+/NADH ratio serves as an energy sensor to regulate mitochondrial metabolism and an increase in the NAD+:NADH ratios activates SIRT1, which increases expression of PGC-1α to promote transcription of TFAM to increase mtDNA replication and mitochondrial biogenesis. Previous studies confirmed that NR activates the PGC-1α-TFAM pathway, leading to mitochondrial biogenesis and increased mtDNA content in cells, including in SMCs with decreased *Fbn1* expression to model Marfan syndrome^44^. Despite increased Pgc-1α and Tfam levels in *Acta2*^*R179C/+*^ SMCs treated with NR, there were no increases in mitochondrial mass, mtDNA, or transcript levels of mitochondrial ETC complex subunits. Rather, NR-mediated increases in complex I activity, potentially through increased protein levels of complex IV subunit mt-Co1, underlies the increase in OXPHOS. There is an established role of an assembled complex IV in maintaining complex I stability and activity in mitochondria^45^. Complexes I, III, and IV assemble to form a supercomplex which is thought to optimize electron transport efficiency, and complex IV contributes to the stability of this supercomplex^46^. Loss of complex IV compromises the mitochondrial membrane potential and reduces total respiration^46^. Thus, stabilization of the mitochondrial supercomplex respirasome appears to underlie the enhanced OXPHOS observed in the NR-treated *Acta2*^*R179C/+*^ SMCs^45^.

Our study shows that NR attenuates cerebrovascular disease associated with the *Acta2* R179C mutation after LCAL by not only decreasing occlusive lesions in the injured carotid artery, but also increasing cerebral blood flow and decreasing deaths due to stroke. In C57BL/6 mice, the posterior communication arteries (PComAs) are bilateral in 21%, unilateral in 53%, and absent in 26%. Mice that underwent middle cerebral artery occlusion with microfilament and had a complete CoW exhibited significantly smaller infarct sizes than mice with incomplete CoWs^47–49^. Notably, 28% of *Acta2*^*SMC-R179C/+*^ mice die post-LCAL, nearly matching the frequency of absent PComAs in C57BL/6 mice, suggesting the lack of PComAs for collateral blood flow may contribute to the stroke-related deaths post-LCAL in the mutant mice.

In *Acta2*^*SMC-R179C/+*^ mice injured with LCAL, NR decreases collaterals that form around the injured carotid artery and reverses ipsilateral leptomeningeal collateral remodeling in the brain, both indicative of improved blood flow. The outward remodeling of the leptomeningeal collaterals in the left cerebral hemispheres of *Acta2*^*SMC-R179C/+*^ mice post-LCAL likely provides compensatory CBF and is potentially triggered by the narrowed CoW collaterals distal to the LCAL in the *Acta2*^*SMC-R179C/+*^ mice. *ACTA2* p.Arg179 patients with MMD-like disease similarly have increased tortuosity of the smaller distal arterial branches of the large cerebral arteries, most likely secondary to remodeling to increase CBF^10^. Notably, a study assessing leptomeningeal collateral status in idiopathic MMD patients demonstrated that poor leptomeningeal collateral status correlated with significantly higher infarction rate and post-operative strokes^50^. Potentially, the reduced narrowing of the distal ICA in NR-treated *Acta2*^SMC-R179C/+^ mice partially restores the blood flow through the CoW to improve compensatory CBF ipsilateral to the LCAL injury in *Acta2*^*SMC-R179C/+*^ mice, thus rendering extensive leptomeningeal collateral remodeling unnecessary.

Induced pluripotent stem cells (iPSCs) from controls and patients with heterozygous *ACTA2* p.R179 pathogenic variants were used to characterize the disrupted differentiation of NCCs to vascular SMCs. These studies identified that SMA localization to the nucleus increases with differentiation from NCCs to SMCs in the control iPSCs, and that levels of nuclear SMA were greatly reduced at all stages of differentiation in *ACTA2* p.R179 cells^20^. Similar to *Acta2*^*R179C/+*^ SMCs, iPSC-derived SMCs from SMDS patients show a lack of differentiation markers, retention of pluripotency markers, and progenitor cell-like properties, including increased proliferation and migration^20^. Further supporting that the MMD-like cerebrovascular in SMDS patients is due to disrupted differentiation of NCCs to SMCs is a report of a *de novo* single nucleotide variant in *MIR145* in a patient with SMDS-like cerebrovascular disease^51^. miRNA 145 is established to suppress stem cell pluripotency markers, such as *OCT4*, *SOX2*, and *NANOG*, and increases levels of SMA in SMCs; thus, disruption of this miRNA likely causes MMD-like cerebrovascular disease through a similar mechanism to *ACTA2* p.R179 variants^51,52^. Other MMD genes have been identified in components of chromatin remodeling complexes, including *YY1AP1, CHD4*, *CNOT3*, and *SETD5*^6^. In the nucleus, SMA associates with the INO80 chromatin remodeling complex, and the R179 variants disrupt this association^20^. Therefore, disruption of nuclear epigenetic changes required for SMC differentiation, leading to highly migratory SMCs, may be a shared mechanism among genetic variants that predispose to MMD. If so, continuance of stem cell features in SMCs, including maintaining glycolysis, may be a common defect amenable to therapy in patients with MMD due to other genetic triggers.

Pulmonary complications of SMDS include pulmonary arterial hypertension (**PAH**), and the histopathology of PAH is characterized by extensive SMC-rich neointimal proliferation, similar to that seen in the LCAL-injured *Acta2*^*SMC-R179C/+*^ mice^53^. Similarly, PAH is associated with defects in mitochondrial metabolism and increased reliance on glycolysis precipitated by hypoxia, and modulation of mitochondrial metabolism in attenuating PAH is currently under investigation^54,55^. Therefore, NR has the potential to also prevent or attenuate other vaso-occlusive manifestations, including PAH, in SMDS patients.

These studies provide critical insight into the molecular pathogenesis of the MMD large artery occlusive lesions in patients with SMDS. We defined an altered phenotype of the *Acta2*^*R179C/+*^ SMCs that is characterized by incomplete differentiation of NCCs to SMCs and the retention of stem cell features, including increased glycolysis and migration. Neurosurgical procedures to restore cerebral perfusion remain the mainstay treatment for MMD patients. We identify here a therapy that increases OXPHOS by providing a precursor of NAD+, which drives quiescence of the SMCs and may prevent MMD lesions in the cerebrovascular arteries. Impaired oxidative metabolism and altered mitochondrial respiration have also been associated with aortic aneurysm formation, and NR treatment prevented aortic aneurysm progression in a mouse model of thoracic aortic disease^56^. Therefore, NR has the potential to also suppress aortic growth in SMDS patients. The safety and tolerability of NR have been confirmed in numerous clinical trials for various conditions, and benefits of NR treatment have been reported^57–62^. We also mimicked a ketogenic diet *in vitro* by maintaining *Acta2*^*R179C/+*^ SMCs in galactose rather than the glucose media and were able to similarly boost OXPHOS and quiescence of the mutant SMCs, suggesting a ketogenic diet as an alternative treatment to prevent cerebrovascular disease in SMDS patients. In summary, modulation of the metabolic pathways in *Acta2*^*R179C/+*^ SMCs towards OXPHOS and differentiation into a quiescent and contractile phenotype has the potential to effectively and safely attenuate MMD-like cerebrovascular disease and improve outcomes for patients with SMDS.

## MATERIALS AND METHODS

### Generation of mouse model

A mutant mouse model (*Acta2*^*SMC-R179C/+*^) with a constitutive, SMC-specific insertion of the heterozygous *Acta2* R179C (c.535C>T) mutation and corresponding WT mice were engineered, as described previously^18^.

### In vivo NR treatment

At 8 weeks of age, the WT and *Acta2*^*SMC-R179C/+*^ mice were treated with either saline or nicotinamide riboside chloride (NR). For NR treatment, NR was dissolved in saline and administered through intraperitoneal injection at a final dose of 1000 mg/kg body weight every other day over a 26-day treatment period beginning five days before the left carotid artery ligation (LCAL).

### Complete left carotid artery ligation injury (LCAL)

All animal studies were performed according to protocols approved by the Institutional Animal Care and Use Committee (IACUC) at the University of Texas Health Science Center at Houston and in accordance with the National Institutes of Health guidelines on the care and safety of laboratory animals. To perform the LCAL injury, WT and *Acta2*^*SMC-R179C/+*^ mice (n=5–7 mice per sex per genotype) were treated as indicated and anesthetized using intraperitoneal injection of 2.5% avertin. The neck was dissected, and the left common carotid artery was exposed. As previously described, the left common carotid artery was completely ligated near its bifurcation with the use of 5–0 silk sutures^63^. The mice were allowed to survive 21 days post-ligation, at which point tissue was collected and imaging studies were performed.

### Histological staining, imaging, and morphological analysis of carotid artery tissue

For morphological analysis of carotid artery tissue, the chest cavities of WT and *Acta2*^*SMC-R179C/+*^ mice (treated and injured as indicated, n=3–6 mice per group) were exposed, the diaphragm cut and the inferior vena cava was severed to drain blood. The mice were then perfused with normal saline and fixed with 10% buffered formalin. Left and right carotid arteries were dissected and isolated with the aorta and fixed in 10% buffered formalin overnight. The samples were dehydrated using standard protocols, and the left and right carotid arteries were paraffin-embedded without further dissection. The entire axial length of the carotid arteries was cut into 5 μm sections from the ligation site to the proximal end of the artery. Carotid artery tissue was stained with hematoxylin and eosin (H&E). Carotid artery tissue was deparaffinized and rehydrated using a series of xylene, ethanol, and PBS washes (100% EtOH, 95% EtOH, 70% EtOH, 1X PBS). The slides were then stained with hematoxylin and eosin (H&E) using standard protocols. Specimens were imaged and photographed using an Olympus microscope. For morphometric analyses, images of stained cross-sections of injured *Acta2*^*SMC*-*R179C/+*^ and WT mice left and right common carotid arteries were analyzed with the ImageJ software (NIH). The lumen area was measured as the total area within the internal elastic lamina. Circumference was measured as the inner arterial circumference. Occlusion percent was measured as the area of intraluminal lesion divided by total lumen area. Medial thickness was measured as an average of three measured distances between the internal and external laminae.

### Immunofluorescence staining, imaging, and analysis of carotid artery tissue

Formalin-fixed paraffin-embedded (FFPE) carotid artery sections from WT and *Acta2*^*SMC-R179C/+*^ mice (n=3–6 mice per group) treated and injured as indicated were de-paraffinized and rehydrated using a series of xylene, ethanol, and PBS washes (100% EtOH, 95% EtOH, 70% EtOH, 1X PBS). Then, the samples were subjected to antigen retrieval using sodium citrate buffer (10 mM sodium citrate, 0.05% Tween 20, pH 6.0) at 95 °C for 30 minutes. The samples were rinsed with cold tap water for 10 min and cooled on ice for 15 min. The tissues were then permeabilized with Tris-buffered saline (TBS) containing 0.03% Triton X-100 and blocked for 1 h with 5% bovine serum albumin (BSA) in TBS at room temperature. The sections were then incubated with primary antibody at 4°C overnight. The next day, the samples were washed with TBS containing 0.01% Tween 20 (TBST) and incubated with appropriate secondary antibodies for 1 hour at room temperature. Following another series of washes, the samples were mounted with ProLong^™^ Diamond Antifade Mountant (Invitrogen), and the slides were allowed to dry for 1 h in the dark. Imaging was performed using a Nikon A1 Confocal Laser Microscope at the UTHealth Center for Advanced Microscopy. Please refer to **Table 2.1** for detailed information on the primary antibodies used. ImageJ (NIH) was used to quantify SMC abundance in intraluminal lesions vs. medial layer by dividing fluorescence of secondary antibody to α-SMA by area of intraluminal lesions or medial layer. ImageJ (NIH) was also used to quantify the number of neovessels per unit area surrounding the left carotid arteries at the site of ligation.

### Histological staining, imaging, and morphological analysis of brain tissue

For morphological analysis of brain tissue, the chest cavities of WT and *Acta2*^SMC-R179C/+^ mice (treated and injured as indicated, n=3–6 mice per group) were exposed, the diaphragm cut, and the inferior vena cava was severed to drain blood. The mice were then perfused with normal saline and fixed with 10% buffered formalin. Brains were harvested and then fixed for an additional 24 h in 10% formalin. The brain was segmented to include the internal carotid artery within the Circle of Willis. The samples were dehydrated using standard protocols, and the brain tissue was paraffin-embedded without further dissection. Paraffin-embedded brain tissue was cut into 5 μm sections. Brain tissue was stained with hematoxylin and eosin (H&E). Brain tissue was deparaffinized and rehydrated using a series of xylene, ethanol, and PBS washes (100% EtOH, 95% EtOH, 70% EtOH, 1X PBS). The slides were then stained with hematoxylin and eosin (H&E) using standard protocols. Specimens were imaged and photographed using an Olympus microscope. For morphometric analyses, images of stained cross-sections of injured *Acta2*^*SMC-R179C/+*^ and WT left and right internal carotid arteries were analyzed with the ImageJ software (NIH). The lumen area was measured as the total area within the internal elastic lamina. Circumference was measured as the inner arterial circumference. Medial thickness was measured as an average of three measured distances between the internal and external laminae.

### Immunofluorescence staining, imaging, and analysis of brain tissue

For morphological analysis of brain tissue, the chest cavities of WT and *Acta2*^*SMC-R179C/+*^ mice (treated and injured as indicated, n=3–6 mice per group) were exposed, the diaphragm cut, and the inferior vena cava was severed to drain blood. The mice were then perfused with normal saline and fixed with 10% buffered formalin. Mice were then perfused with fluorescent tomato lectin to label the vascular endothelium. Brains were harvested and then fixed for an additional 24 h in 10% formalin. For Iba1 and GFAP staining, the cortical surfaces of tomato lectin-stained brains were planed by vibratome and sectioned at 30 μm. Sections were washed with PBS, incubated with blocking buffer (10% goat serum, 0.3% Triton X-100 in PBS), and then incubated overnight at 4 °C with the primary antibody. For detection of the IBA-1 antibody, we used either donkey anti-rabbit IgG-Alexa 594 or 488 (1:200, Thermo Fisher Scientific, MA, USA). Images were obtained using a Leica TCS SPE confocal system and a Leica DMi8 fluorescence microscope system (Leica Biosystem, IL, USA). Multiple images were captured with the 10X objective covering the whole brain or brain hemisphere Sect. (48 images) and stitched to generate a single image (Leica LAS X software). Higher magnification was performed of selected regions using 20X or 40X objectives. Image analysis was performed using ImageJ software (National Institutes of Health).

### In vivo nanoparticle contrast-enhanced computed tomography (n-CECT) imaging

WT and *Acta2*^*SMC-R179C/+*^ mice (n=17–32 per group) were treated and injured as indicated and were anesthetized with isoflurane and injected with liposomal iodine contrast (500 ul; jugular vein). This long-circulating liposomal-iodinated contrast agent that is cleared slowly from the blood was used to visualize and quantify the blood vessels of the head and brain. The liposomal contrast agent was prepared using methods described previously^37^. Within 60 min following contrast injection, mice were imaged by n-CECT to visualize the head and brain vasculature^37^. For imaging, mice were anesthetized with isoflurane (4% induction) and then placed in an imaging cassette within a SkyScan 1276 microCT imaging system (Bruker, Belgium). Mice were then delivered 1.5% isoflurane in 25 % O_2_ (balance N_2_) by face mask. The imaging chamber was warmed to provide heat support during imaging. n-CECT imaging was performed at an applied voltage of 70 kV, current of 200 uA, 200 msec exposure time, and using the aluminum filter (1 mm). Imaging was conducted at 0.3° rotation (1200 projection images), averaging 3 images per projection, to achieve a final spatial resolution of 13 μm. 3D image reconstruction was performed with NRecon (Bruker, Belgium) and exported as DICOM format data sets. Image segmentation and quantitative analysis were performed with Horos 6 (www.Horosproject.org). Vascular morphometric analyses were performed as described previously^37^. Arterial diameters were obtained from 3D reconstructed multiplanar reconstructed (MPR) images. n-CECT imaging was supported by NIH S10OD030336 and performed through the MicroCT Imaging Facility at the McGovern Medical School at UTHealth.

### Imaging and measurement of cerebral blood flow

Laser speckle contrast imaging (LSCI) was performed as previously described in WT and *Acta2*^*SMC-R179C/+*^ mice (n=3–5 mice per group) treated and injured as indicated^64^. Briefly, the skull over the parietal cortex was exposed. LSCI was performed on mice in supine position using a 785 nm laser diode and controller (L785P090 and LDC 205C, Thorlabs) with temperature control (TED200C, Thorlabs, Newton, NJ) and acquisition software (FOIL, Dr. Andrew Dunn11, 12). A monochrome CCD camera (acA-640–120gm, BASLER, Ahrensburg, Germany) equipped with a macro zoom lens (ZOOM 7000, NAVITAR, Rochester, NY) was used to acquire images averaged from sets of fifteen sequential speckle images at 30 second time intervals. MATLAB (MathWorks, Natick, MA) was used to define regions of interest and calculate inverse correlation times (ICT). Relative cerebral blood flow (CBF) was calculated as a ratio of left-sided (ipsilateral to the LCAL injury) to right-sided (contralateral to LCAL injury).

### Lectin staining to assess leptomeningeal collateral remodeling

WT and *Acta2*^*SMC-R179C/+*^ mice (n=6–7 per group) were treated and injured as indicated and were perfused transcardially with heparinized PBS and after, 4% paraformaldehyde. Mice were subsequently perfused with fluorescent tomato lectin (Lycopersicon Esculentum, DyLight 594, Vector Laboratories) to label vascular endothelium. The cortical surface was “planed off” by vibratome to provide contiguous cortical surface vessel preparations containing the distalmost arterioles and the connected leptomeningeal collaterals. Images were obtained using a Leica TCS SPE confocal system and a Leica DMi8 fluorescence microscope system (Leica Biosystem, Richmond, IL, USA). Multiple images of the whole brain or individual hemispheres were captured with the 10× objective and stitched to generate a single high-resolution image (Leica LAS X software ver. 3.6.0.20104, Leica Biosystem, Nussloch, German). The inner diameter of the leptomeningeal collaterals was measured with ImageJ software.

### Microfil perfusion and ex vivo μ-CT imaging

To perform Microfil perfusion of eight week-old WT and *Acta2*^*SMC-R179C/+*^ mouse brains, we utilized a modified version of previously published cerebral contrast perfusion protocols^65,66^. The animals were injected intraperitoneally with three μg acepromazine maleate (Vetoquinol, Lavaltrie, QC, Canada) per gram of body weight for vasodilation and heparin (100 U/ml, 0.2 ml per mouse; #949516, McKesson) for anticoagulation. Heparin was allowed to circulate for 5 min. Mice were anesthetized using intraperitoneal injection of 2.5% avertin. The chest cavity was opened, and the diaphragm was cut to expose the heart, inferior vena cava, liver, and trachea. A 24-gauge needle was then inserted into the abdominal aorta and advanced toward the thoracic descending aorta. The needle was secured with two 5–0 silk suture ligatures. The inferior vena cava was severed immediately before perfusion to provide venous outflow and drain cerebral blood. The needle was connected to a syringe pump (NE-300, New Era Pump Systems, NY). Animals were then perfused with 20 ml of room temperature PBS containing sodium nitroprusside (2 mM; Fluka) and papaverine (150 μM; Sigma-Aldrich) to ensure dilation of all vessels and accessibility to filling solutions with an incremental increase from 1 to 5 ml/min. Perfusion was continued with 20 ml of room temperature 10% buffered formalin at 5 ml/min. The working Microfil solution was prepared by mixing five parts of Microfil MV-122, four parts diluent, and 0.5 parts catalyst, per manufacturer recommendations. Following fixative infusion, freshly prepared Microfil MV-122 compound was loaded and perfused by hand. Yellowing of the nose indicates successful perfusion. The Microfil-perfused mouse was left overnight on ice at 4 C. The brain was removed the next day and stored in 10% buffered formalin at 4 C for at least 24 h prior to μ-CT imaging.

*Ex vivo* micro-computed tomography (μ-CT) imaging was performed on a small animal micro-CT system (Inveon, Siemens Inc., Knoxville, TN, USA) as previously described^37^. In this study, images were acquired at 20 μm isotropic voxel size. The acquired X-ray projection images were reconstructed into 3D datasets using a filtered back-projection reconstruction algorithm on Cobra software (version 6.3.39.0, EXXIM Computing Corporation, Pleasanton, CA, USA). All datasets were calibrated using Hounsfield Units (HU) for image analysis.

### Mouse echocardiography

Transthoracic echocardiography (Vevo 3100 imaging system; MX550D, 40 MHz transducer; VisualSonics, Toronto, Canada) was performed on age-matched and sex-matched WT and *Acta2*^*SMC-R179C/+*^ mice to determine aortic root and ascending aortic diameter *in vivo*. Echocardiography imaging was obtained every two months from two to 12 months of age. The mice were restrained in the supine position, weighed, and anesthetized with 0.6 liters per minute of room air containing 2% isoflurane via nose cone. The heart rate was monitored, and the body temperature was maintained at approximately 37 °C using a heated platform. Two-dimensional echocardiography images were recorded in B-mode and analyzed using a Vevo 3000 Ultrasound Machine equipped with a 40 MHz ultrasonic linear probe (VisualSonics, Toronto, Ontario, Canada). Images were obtained in the parasternal long-axis view, and aortic measurements were made in at least three separate heartbeats per mouse in late diastole. Three measurements of maximal internal diameter at the aortic root and ascending aorta were obtained. The data were analyzed by an operator blinded to the treatment groups.

### Mouse scRNA-seq and analysis

Tissues were isolated from eight week-old female *Acta2*^*SMC-R179C/+*^ and WT mouse littermates, pooled together, and digested to obtain single-cell suspensions as previously described^67^. Viable single cells were detected and collected using flow cytometry. Barcoded cDNA was generated using a Chromium Single Cell 3′ v2 Reagent Kit (10x Genomics). This step was followed by cDNA amplification, truncation, and library preparation. A NovaSeq 6000 Next Generation Sequencing system (Illumina) was used to perform sequencing at the Baylor College of Medicine Single Cell Genomics Core. Data files from previously reported scRNA-seq data from ascending aortic/transverse arch tissue from six mice were analyzed. Raw sequencing data were demultiplexed and aligned to the mm10 mouse genome using the CellRanger v.6.0.0 scRNA analysis pipeline (10X Genomics). Data from control and *Acta2*^SMC-R179C/+^ were merged into a single object and analyzed in parallel using the Seurat package (v.4.1.0) in R. Following standard quality control metrics to remove doublets and debris, data were normalized, scaled, and regressed to read depth. Principal component analysis, nonlinear dimensional reduction, and unsupervised clustering at a pre-set resolution of 0.3 were performed. SMC subsets were selected manually for downstream analyses. Differential gene expression (DEG) testing was performed in Seurat using the “FindMarkers” function to perform Wilcoxon rank-sum testing. Genes with an average [log2Fold-change(FC)] > 0.25 and adjusted p-value <0.05 were considered significant. Raw and processed sequencing data are available through the Gene Expression Omnibus (GEO) repository (GEO accession # GSE201091). scRNA-seq data were analyzed in the Seurat package using ‘sctransform’ based normalization of the control and *Acta2*^SMC-R179C/+^ samples followed by cell clustering with the resolution parameter set at 0.3.

### Mouse aortic SMC isolation and culture

SMCs were explanted from the ascending aortas of eight week-old age- and sex-matched WT and *Acta2*^*SMC*-*R179C/+*^ mice as previously described^68^. SMCs were cultured in Smooth Muscle Basal Media (SmBm, Promo Cell) supplemented with 20% FBS (Gibco), insulin, epidermal growth factor, fibroblast growth factor (Promo Cell), HEPES (Millipore Sigma), sodium pyruvate (Millipore Sigma), L-glutamine (Millipore Sigma), and antibiotic/anti-mycotic (Millipore Sigma).

### *In vitro* nicotinamide riboside treatment

SMCs were treated with either 0.25 mg/ml nicotinamide riboside triflate (NR) (Carbosynth Biosynth) or ddH_2_O for five days. Basal media containing NR or ddH2O was added/changed to SMCs on day(s) 1, 3, and 5. SMCs were then serum-starved in SmBM containing 1% FBS, HEPES, sodium pyruvate, L-glutamine, and antibiotic/anti-mycotic with either NR or ddH_2_O for 24 hours unless otherwise noted. Cell culture assays were performed in triplicate, and the data shown are representative of at least three experiments.

### Quantitative real time-PCR (qRT-PCR) and mtDNA quantification

Total RNA was isolated from cultured WT and *Acta2*^SMC-R179C/+^ SMCs treated as indicated (Purelink RNA kit, ThermoFisher), quantified by Nanodrop (Thermo Fisher Scientific), and 50 ng of RNA from each sample was reverse transcribed for cDNA synthesis using QScript reagent (Quantabio) using PCR cycling of 25 °C for five min, 42 °C for 2 h, and 85 °C for 5 min, holding at 4 °C. Quantitative real time-polymerase chain reactions (qRT-PCR) were performed, and mRNA expression was assessed using SYBR green chemistry (primers listed in **Table 3.1** below, reaction master mix from Quantabio) or Taqman probes (Taqman probes were purchased from Applied Biosystems, qPCR master mix obtained from Takara Biosciences). Taqman chemistry was used for quantifying contractile gene transcripts (Applied Biosciences) and SYBR Green (Millipore Sigma) for all other genes using appropriate master mixes (Quantabio). Reactions were performed in triplicate and *Gapdh* and *Pp1a* were used as endogenous controls for SYBR and Taqman reactions in SMCs, respectively. For analysis of mitochondrial DNA (mtDNA) levels, total DNA from cells and tissues was extracted with the DNEasy Blood and Tissue kit (Qiagen) according to manufacturer guidelines. DNA was amplified using primers for mtDNA-specific *mt-Co1* (mitochondrial-encoded cytochrome *c* oxidase 1) and *mt-Nd1* (mitochondrial-encoded NADH dehydrogenase 1), then normalized to *Pp1a* (protein phosphatase 1a). The samples underwent thermal cycling using the LightCycler 96 (Roche) at 95 °C for 10 minutes, then 40 cycles of 95 °C for 15 seconds and 60 °C for 1 minute. Data were analyzed using the Light Cycler 96 software (Roche), and the ΔΔCT method was utilized to compute relative gene expression.

### Immunoblotting and analysis

Explanted WT and *Acta2*^*SMC-R179C/+*^ SMCs were treated as indicated. SMCs were lysed in 50–100 μL of RIPA buffer (50 mM Tris-HCl pH 7.4, 1% NP40, 0.25% Na-deoxycholate, 150 mM NaCl, 1 mM EDTA, 1 mM PMSF) supplemented with 30 μL/mL protease (P8340, Millipore Sigma) and 10 μL/mL phosphatase inhibitor cocktails 2 (P5726, Millipore Sigma) and 3 (P0044, Millipore Sigma) after incubation at 4 °C for 30 min. Samples were then sonicated for 20 seconds. Protein concentration was quantified using Bradford assay (Bio-Rad Laboratories). Equivalent amounts (between 3 and 30 μg depending upon the primary antibody used) were boiled at 100 °C for five min in loading buffer. Subsequently, the protein was loaded and run on 4–20% TGX SDS-PAGE gels for ~3 h at 60 V (Bio-Rad Laboratories) and transferred to PVDF membranes (Millipore Sigma) for overnight transfer at 30 V. After transfer, PVDF membranes were blocked with 5% non-fat dry milk or 5% BSA in TBST for one hour and incubated overnight with primary antibodies at a concentration of 1:1000 at 4 °C. Following overnight incubation, the blots underwent a series of washes and incubation with corresponding secondary antibodies at a concentration of 1:4000 for one hour at room temperature. Lamin A/C and H3 are used as loading controls, as Gapdh is an enzyme utilized in glycolysis and may have altered expression in mutant SMCs. Bands were visualized by chemiluminescent substrate (Bio-Rad) using the Bio-Rad ChemiDoc Imaging system. ImageJ software was used to quantify band intensities. Please refer to **Table 2.1** for detailed information on antibodies.

### BrdU proliferation assay

Explanted WT and *Acta2*^*R179C/+*^ SMCs were treated where indicated and seeded at a density of 5,000 cells per well in triplicates in a 96-well plate for attachment overnight. The cells underwent BrdU incorporation ELISA using a standardized protocol kit (Cell Signaling Technologies). Briefly, the cells were subsequently incubated with a final concentration of 10 μM BrdU for 24 h. The cells were washed, fixed and denatured, and incubated in 1X detection antibody for 1 h at room temperature. After a series of washes, the cells were incubated in 1X HRP-conjugated secondary antibody solution for 30 min at room temperature. After another series of washes, TMB substrate was added, and cells were incubated for 10 min. Then STOP solution was added and absorbance was read at 450 nm. Analysis of proliferation was performed with ELISA according to the manufacturer’s instructions (Cell Signaling Technologies). Assays were performed in triplicate, and graphs are representative of at least three independent experiments.

### Transwell migration assay

A modified Boyden chamber assay was used to quantify cell migration. Explanted WT and *Acta2*^*R179C/+*^ SMCs were treated as indicated and then seeded at a density of 25,000 cells per well in triplicate. Cells were seeded in chambers containing a permeable membrane with a pore size of 8 × 10^−3^. Chambers were then submerged in a basal media containing well (~1 ml), allowing cells to attach and migrate overnight. Following this incubation, the membranes containing the migratory cells were washed with PBS, methanol, and distilled water. The cells were then stained with NucBlue (Thermo Fisher Scientific). The membranes containing the migrated cells were excised and mounted on glass slides. Stained membranes were photographed by a blinded observer in 4 random 400x fields using filters for DAPI on a Zoe Fluorescent Cell Imager (Bio-Rad Laboratories). Migrated cells were quantified using ImageJ software. Assays were performed in triplicate, and graphs are representative of at least three independent experiments.

### SMC immunostaining and confocal microscopy

WT and *Acta2*^*R179C/+*^ SMCs were treated as indicated, seeded at a density of 15,000 cells per 22 mm round coverslip in 6-well plates, and allowed to attach overnight. Following, coverslips were washed with 1X PBS and fixed with 4% paraformaldehyde in 1X PBS. Cells were permeabilized in 0.3% Triton X-100 for 15 min, blocked in 1% BSA in PBS supplemented with 0.5% Tween 20 for 1 h at room temperature, and then incubated with 1:100 dilution of primary antibody at 4 °C overnight. After a series of washes, cells stained for α-SMA were incubated with AlexaFluor 488 Goat-anti-mouse at room temperature for 1 h and Texas Red^™^-X Phalloidin for 30 min in the dark, then mounted in VectaShield with DAPI (ThermoFisher). Immunofluorescent images were obtained using the Nikon A1-R confocal microscope.

### Transmission electron microscopy and mitochondrial morphological assessment

WT and *Acta2*^*R179C/+*^ SMCs were treated as indicated and then isolated and stored in 3% glutaraldehyde in PBS at 4 C until tissue processing. Samples were washed in 1M phosphate buffer (pH 7.3) post-fixed in 1% osmium tetroxide for 1 h and dehydrated through a series of graded alcohol. Samples were infiltrated with acetone and Epon 812 plastic resin and embedded in plastic molds with 100% Epon 812 plastic resin. Thick sections (1 μm) were cut from the Epon 812 blocks with a Leica EM UC7 ultra-microtome, mounted on glass slides, and stained with Toluidine Blue. Toluidine Blue stained slides were placed on an Olympus BX53F2 microscope and images were captured with an Olympus DP27 camera at 10X magnification to confirm appropriate cell preservation for ultrastructural imaging and assessment. Ultra-thin sections (70–80 nm) were cut from Epon 812 blocks with a Leica EM UC7 ultra-microtome, mounted on 100 mesh copper grids, and stained with 2% uranyl acetate and Reynold’s lead stain. Grids were placed on a JEOL JEM-1230 electron microscope and images were captured with an AMT XR80 digital camera at 1500x, 2500x, and 5000x. All images were obtained at the Texas Heart Institute for examination under non-GLP conditions. Ultrasound images at 2500x magnification were evaluated for the number of mitochondria in one cell (n=10 images showing an entire cell per group). Areas of cross-sectional images of random mitochondria were obtained from ultrastructural images at 2500x magnification (n=10 images showing an entire cell per group). The mitochondrial morphologic assessment included the evaluation of irregular cristae at 5000x magnification (n=10 images per group). All parameters were evaluated using a semi-quantitative grading (0=absent, 1=mild, 2=moderate, 3=marked). All ultrastructural parameters were assessed blinded to the type of samples.

### MitoTracker Deep Red (MTDR) staining and live cell imaging

Mitochondrial mass/function levels were quantified using flow cytometry. WT and *Acta2*^*R179C/+*^ SMCs were treated as indicated and plated at a density of 100,000 cells into 6-well plates. Cells were trypsinized and labeled with MTDR (10 nM) (M22426, Invitrogen) for 15 minutes at 37 °C, 5% CO_2_. Probes were washed out using PBS, and cells were resuspended in PBS for reading on a flow cytometer. Using an LSRFortessa flow cytometer (Becton Dickinson, Franklin Lakes, NJ, USA), 10,000 cells were acquired (FACSDiva software, BD FACSDiva v8.0.1, Becton Dickinson), and the data were analyzed using the single cell analysis software FlowJo. Assays were performed in triplicate, and graphs are representative of at least three independent experiments.

### MitoTracker Deep Red staining and flow cytometry

Mitochondrial mass/function levels were quantified using flow cytometry. Vehicle- and NR-treated WT and *Acta2*^*R179C/+*^ SMCs were plated at a density of 100,000 cells into 6-well plates. Cells were trypsinized and labeled with MitoTracker Deep Red (10 nM) (M22426, Invitrogen) for 15 minutes at 37 C, 5% CO_2_. Probes were washed out using PBS, and cells were resuspended in PBS for reading on a flow cytometer. Using an LSRFortessa flow cytometer (Becton Dickinson, Franklin Lakes, NJ, USA) 10,000 cells were acquired (FACSDiva software, BD FACSDiva v8.0.1, Becton Dickinson), and the data were analyzed using the single cell analysis software FlowJo.

### Quantification of lactate generation

WT and *Acta2*^*R179C/+*^ SMCs were treated as indicated. L-lactate was quantified using a commercially available colorimetric L-lactate kit according to the manufacturer’s instructions (L-Lactate Assay kit Colorimetric, ab65331, Abcam, Cambridge, Massachusetts, USA). The reaction was started by adding the reaction mix to the sample wells and incubation for 30 minutes. L-Lactate levels were measured at 450 nm. Assays were performed in triplicate, and graphs are representative of at least three independent experiments.

### Complex I activity assay

The activity of Complex I was analyzed using the Complex I Enzyme Activity Assay Kit according to the manufacturer’s instructions (Abcam, ab109721). Briefly, mitochondria were isolated (Mitochondria Isolation Kit, ab110168) from vehicle- and NR-treated WT and *Acta2*^*R179C/+*^ SMCs, and mitochondrial protein concentrations were measured using the Bradford Assay. Five μg of protein were combined with Incubation Solution (Abcam). Each sample was loaded in triplicate (200 μL/well) and incubated for 3.5 h at room temperature. Complex I activity was determined by following changes in 450 nm absorbance every 5 min for 1 h following the addition of Assay Solution to the wells (abcam). Assays were performed in triplicate, and graphs are representative of at least three independent experiments.

### Extracellular flux analyses

Extracellular flux analyses were performed to assess the metabolic profiles of cells. WT and *Acta2*^*R179C/+*^ SMCs were treated as indicated and seeded at a density of 25,000 cells in SmBm supplemented with 20% FBS, insulin, epidermal growth factor, fibroblast growth factor, HEPES, sodium pyruvate, L-glutamine, and antibiotic/anti-mycotic overnight. Oxygen consumption rate (OCR) and extracellular acidification rate (ECAR) were measured in XFp Extracellular Flux Analyzers (Seahorse Biosciences) utilizing the Mito Stress Test kit (Seahorse Biosciences) and its associated standardized protocol. SmBm media was replaced by Seahorse DMEM assay media (1 mM pyruvate, 2 mM glutamine, and 10 mM glucose). Three measurements were obtained under basal conditions and on addition of oligomycin (1 μM), fluoro-carbonyl cyanide phenylhydrazone (1.5 μM), and rotenone + antimycin A (1μM). Hoechst stain was added at the end of the assay at a final concentration of 2 μM. Hoechst-stained cells in the wells were imaged and counted with the LionHeart Imager. OCR and ECAR measurements were normalized to cell count. Assays were performed in triplicate, and graphs are representative of at least three independent experiments.

### Apoptosis/necrosis assay

Apoptosis and necrosis were quantified using flow cytometry. A GFP-CERTIFIED^®^ Apoptosis/Necrosis Detection Kit (Enzo Life Sciences) was used to determine the effect of NR on SMC apoptosis, as per manufacturer’s instructions. WT and *Acta2*^*R179C/+*^ SMCs were plated on 6-well plates and treated as indicated. On the day of the assay, positive control cells were treated with staurosporine at a final concentration of 2 μM. Cells were collected by trypsinization followed by centrifugation and then stained with apoptosis detection reagent for 5 minutes. Using an LSRFortessa flow cytometer (Becton Dickinson, Franklin Lakes, NJ, USA), 10,000 cells were acquired (FACSDiva software, BD FACSDiva v8.0.1, Becton Dickinson), and the data were analyzed using the single cell analysis software FlowJo.

### Measurement of intracellular mitochondrial ROS levels

Intracellular mitochondrial ROS levels were quantified using flow cytometry. WT and *Acta2*^*R179C/+*^ SMCs were treated as indicated, seeded at a density of 100,000 cells in a 6-well plate, and trypsinized and labeled with MitoSox Red (5 μM) for 30 minutes. Probes were washed out using PBS, and cells were resuspended in PBS for reading on a flow cytometer. Using an LSRFortessa flow cytometer (Becton Dickinson, Franklin Lakes, NJ, USA) 10,000 cells were acquired (FACSDiva software, BD FACSDiva v8.0.1, Becton Dickinson), and the data were analyzed using the single cell analysis software FlowJo. Assays were performed in triplicate, and graphs are representative of at least three independent experiments.

### Measurement of mitochondrial membrane potential

The positive control was treated with carbonyl cyanide 3-chlorophenylhydrazone (CCCP) at a final concentration of 50 μM and incubated at 37 C, 5% CO_2_ for 5 min. Vehicle- and NR-treated WT and *Acta2*^*R179C/+*^ SMCs and the positive control were incubated with JC-1 fluorescent dye (Invitrogen, CA, USA) at a concentration of 2 μM for 20 min at 37 C, 5% CO_2_, and then subsequently washed with PBS. The mitochondrial membrane potential was detected by flow cytometry for quantification of green and orange-red emissions. A red fluorescent JC-1 signal is indicative of healthy cells with a high △Ψm, whereas a green fluorescent JC-1 signal is indicative of unhealthy cells with a low △Ψm. Assays were performed in triplicate and graphs are representative of at least three independent experiments.

### Metabolomics

WT and *Acta2*^*R179C/+*^ cells were treated as indicated, and targeted metabolomics experiments were performed at the MD Anderson Cancer Center Metabolomics Facility. Analysis of polar metabolites was performed by ion chromatography – high resolution mass spectrometry (IC-HRMS). To determine the incorporation of glucose and glutamine carbon into the glycolysis pathway, intracellular tricarboxylic acid (TCA) cycle and pentose phosphate pathway nucleotides extracts were prepared and analyzed by HRMS. Cells were washed with PBS before incubating in fresh medium containing 10 mM ^13^C_2_-Glucose for 4 and 24 h or 2 mM ^13^C_5_-Glutamine for 24 h. Cells were quickly washed with ice-cold deionized water with 80% ammonium bicarbonate to remove extra medium components. Metabolites were extracted using cold 80/20 (v/v) methanol/water with 0.1% ammonium hydroxide. Samples were centrifuged at 17,000 g for 5 min at 4 C, and supernatants were transferred to clean tubes, followed by evaporation to dryness under nitrogen. Samples were reconstituted in deionized water, then 5 μl was injected into a Thermo Scientific Dionex ICS-6000+ capillary ion chromatography (IC) system containing a Thermo IonPac AS11 250×2 mm 4 μm column. IC flow rate was 360 uL/min (at 30°C) and the gradient conditions are as follows: started with an initial 1mM KOH, increased to 35 mM at 25 min, then to 99 mM at 39 min., held 99 mM for 10 mins. The total run time is 50 min. To assist the desolvation for better sensitivity, methanol was delivered by an external pump and combined with the eluent via a low dead volume mixing tee. Data were acquired using a Thermo Orbitrap IQ-X Tribrid Mass Spectrometer under ESI negative mode. Then the raw files were imported to Thermo Trace Finder software for final analysis. The fractional abundance of each isotopologue is calculated by the peak area of the corresponding isotopology normalized by the sum of all isotopoloy areas. (Du, D. et al., *BMC Bioinformatics*,2019). The relative abundance of each metabolite was normalized by total peak intensity.

### Statistical analysis and reproducibility

Nonparametric statistical tests were conducted for all mouse studies. For two groups, an unpaired Mann–Whitney analysis was performed. For three or more groups, the Kruskal–Wallis analysis was performed with Dunn post-tests to compare between specific groups. Cell culture data were analyzed using Student *t* tests, 1-way ANOVA, or 2-way ANOVA, both 2-tailed and unpaired. For all experiments except single-cell RNA sequencing, a minimum of three biological replicates were performed. RT-PCR, blot quantitation, and imaging quantitation results with two groups were analyzed by one-way ANOVA. RT-PCR results and blot quantitation results with three or more groups were analyzed by two-way ANOVA followed by Tukey’s multiple comparisons test. Data representation and statistical analysis were performed using GraphPad Prism software. All data are shown as mean ± standard deviation (SD). Error bars on all mouse data represent standard deviation. Data were tested for normality using GraphPad Prism software version 9.4.0 (Graph Pad Software, Inc., San Diego, CA). Data representation and statistical analysis were performed using GraphPad Prism software.

## Supplementary Material

1

## Figures and Tables

**Table 1:** Primers used in this study. qRT-PCR; quantitative real time-polymerase chain reaction. ChIP; chromatin immunoprecipitation. mtDNA; mitochondrial DNA.

Target (qRT-PCR)	Primers
*Acta2*	fwd: 5’-CACTGTCAGGAATCCTGTGA-3’ rvs: 5’-CAAAGCCGGCCTTACAGA-3’
*Actg2*	fwd: 5’-CCGCCCTAGACATCAGGGT-3’ rvs: 5’-TCTTCTGGTGCTACTCGAAGC-3’
*Cnn1*	fwd: 5’-GTCCACCCTCCTGGCTTT-3’ rvs: 5’-AAACTTGTTGGTGCCCATCT-3’
*Gapdh*	fwd: 5’-TGAAGGTCGGAGTCAACGGA-3’ rvs: 5’-GGTCAGGTCCACCACTGACAC-3’
*Mt-Co1*	fwd: 5’-CTCGCCTAATTTATTCCACTTCA-3’ rvs: 5’-GGGGCTAGGGGTAGGGTTAT-3’
*Mt-Nd1*	fwd: 5’-CTAGCAGAAACAAACCGGGC-3’ rvs: 5’-CCGGCTGCGTATTCTACGTT-3’
*Myh11*	fwd: 5’-AGATGGTTCTGAGGAGGAAACG-3’ rvs: 5’-AAAACTGTAGAAAGTTGCTTATTCACT-3’
*Pp1a*	fwd: 5’-ACGCCACTGTCGCTTTTC-3’ rvs: 5’-GCAAACAGCTCGAAGGAGAC-3’
*Tagln*	fwd: 5’-TCTTTGAAGGCAAAGACATGG-3’ rvs: 5’-TTATGCTCCTGCGCTTTCTT-3’
Target (ChIP)	Primers
*Acta2* (mouse)	fwd: 5’-AGAGTGAACGGCCAGCTTCA-3’ rvs: 5’-AGGCTGAACGCTGAAGGGTT-3’
*Cnn1* (mouse)	fwd: 5’-CCAGATGAGAGCTGTCTAGATCT-3’ rvs: 5’-GCCAGGTTAACAGGTCTTGG-3’
*Myh11* (mouse)	fwd: 5’-GGCCTTTTTGGGTTGTCTCC-3’ rvs: 5’-CCTTGCACACACACCACTCA-3’
*Tagln* (mouse)	fwd: 5’-CCAAGTCCGGGTAACAAGGAA-3’ rvs: 5’-GCATGCTTTGGAGATGCTGC-3’
Target (mtDNA)	Primers
*Mt-Co1*	fwd: 5’-CTCGCCTAATTTATTCCACTTCA-3’ rvs: 5’-GGGGCTAGGGGTAGGGTTAT-3’
*Mt-Nd1*	fwd: 5’-CTAGCAGAAACAAACCGGGC-3’ rvs: 5’-CCGGCTGCGTATTCTACGTT-3’

**Table 2:** Antibodies used in this study. CST; Cell Signaling Technologies. SC; Santa Cruz. WB; Western blot. ChIP; chromatin immunoprecipitation. IF; immunofluorescence staining.

Antigen	Company, Product #	Use in paper
αSMA	Sigma A5228	WB, IF (cells)
αSMA	Abcam ab21027	IF (tissue)
Calponin	Abcam ab46794	WB
CD31/PECAM-1	Novus Biologicals NB100–2284	IF (tissue)
Gapdh	CST 2118	WB
Histone H3	CST 4499	WB
F4/80	MF48000	IF (tissue)
GFAP	53-9892-80 (Invitrogen)	IF (tissue)
Iba1	019–19741 (Fujifilm)	IF (tissue)
Lamin A/C	CST 4777	WB
Pgc-1α	Abcam ab191838	WB
SM-MHC	Abcam ab125884	WB
SM-22α (Transgelin)	Abcam ab14106	WB
Tfam (m-Tfa)	Abcam ab47517	WB
Total OXPHOS Rodent	Abcam ab110413	WB
