# Supplementary material for: Augmenting Mitochondrial Respiration in Immature Smooth Muscle Cells with an ACTA2 Pathogenic Variant Mitigates Moyamoya-like Cerebrovascular Disease
Source: Res Sq. 2023 Oct 12:rs.3.rs-3304679. Preprint. [Version 1] doi: 10.21203/rs.3.rs-3304679/v1 (PMC10602100; doi:10.21203/rs.3.rs-3304679/v1)
Supplement: 1 [file NIHPPRS3304679V1-supplement-1.pdf]

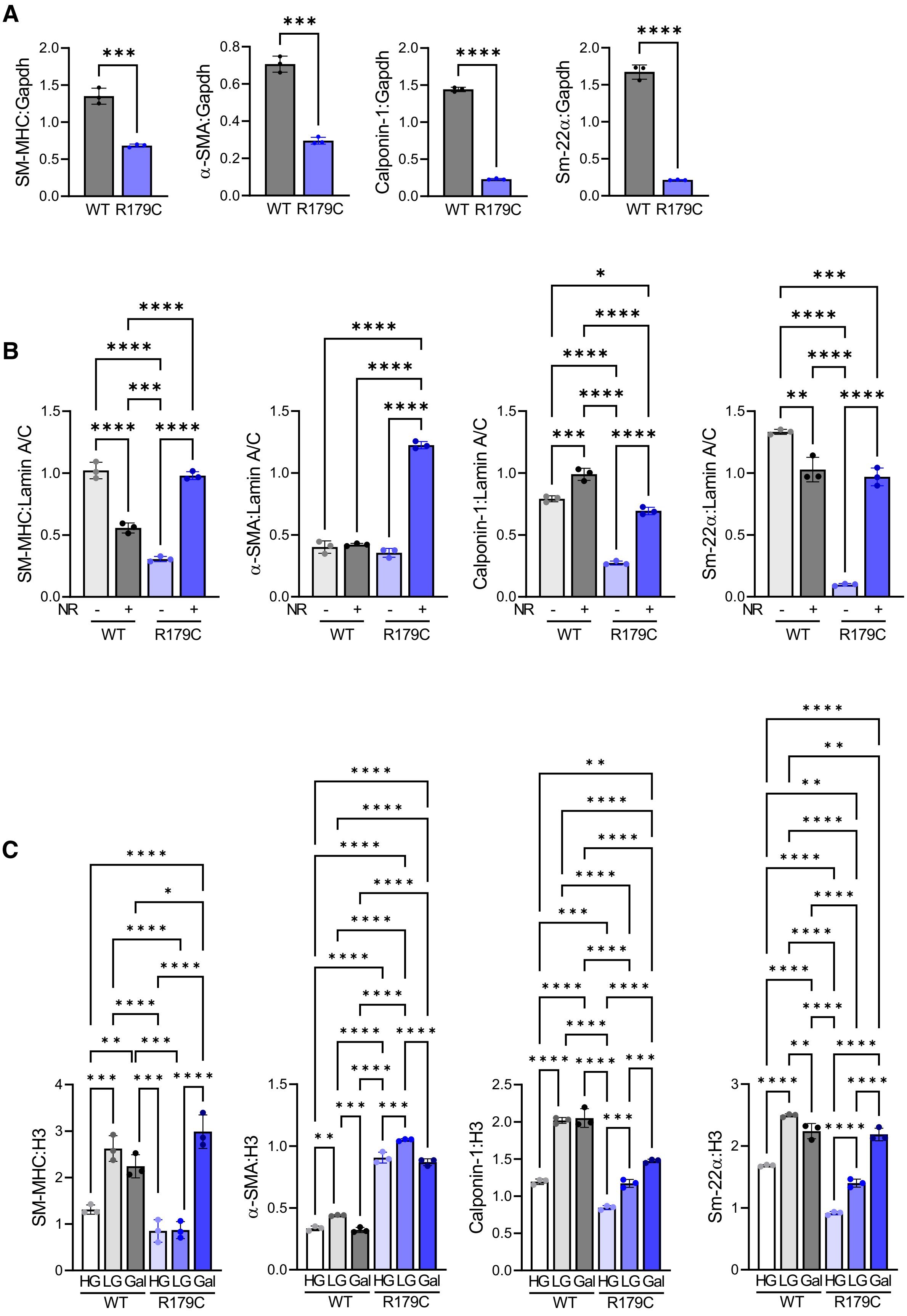

Supplemental 1. A. Western blot band quantification for Fig. 1A. **B.** Western blot band quantification for Fig. 2B. **C.** Western blot band quantification for Fig. 2F.

930

A

B

C

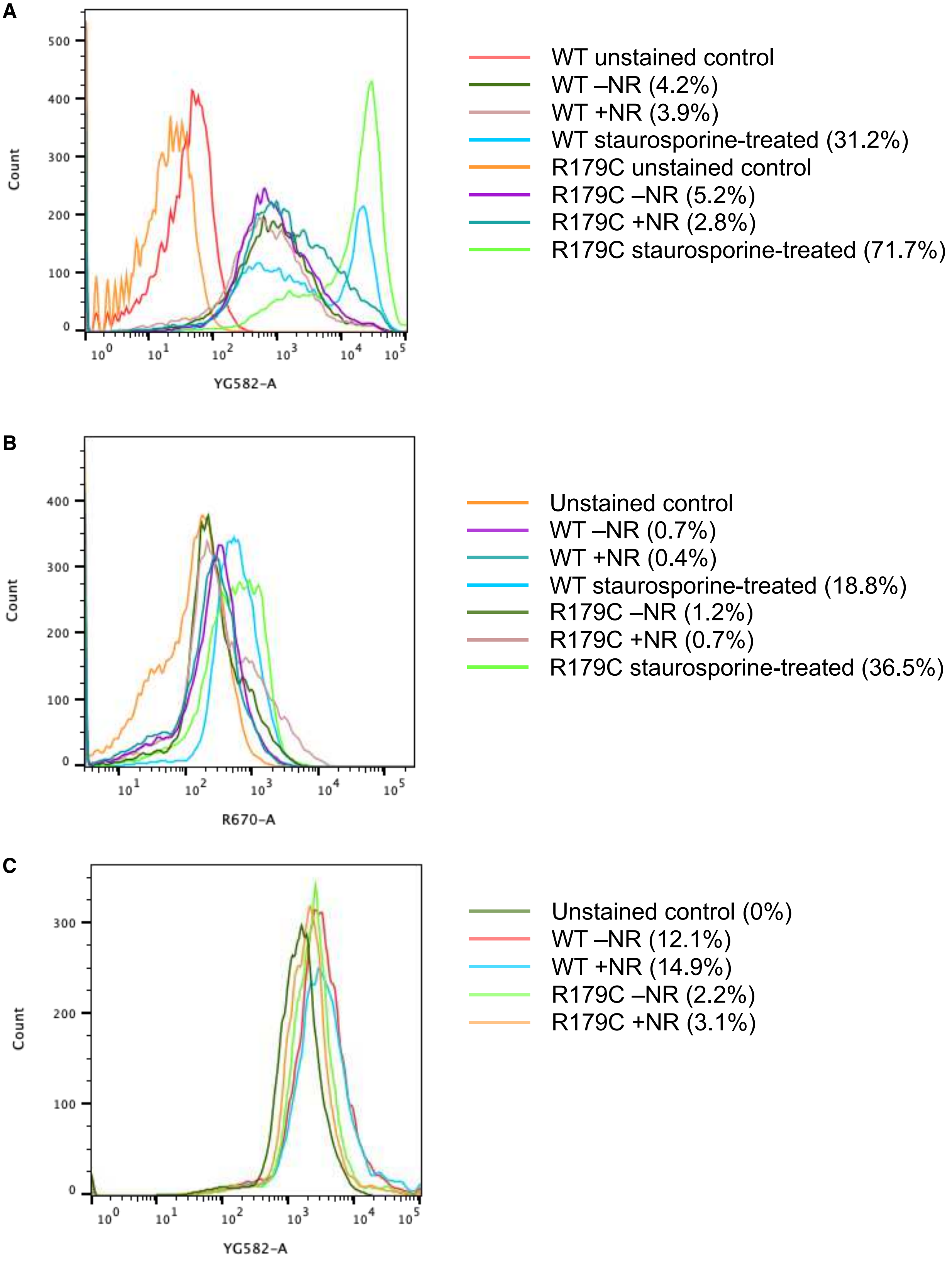

Supplemental 2. NR does not affect apoptosis (**A**) or necrosis (**B**) in WT or *Acta2*$^{R179C/+}$ SMCs. **C.** NR does not affect mitochondrial ROS formation in WT or *Acta2*$^{R179C/+}$ SMCs. NR; nicotinamide riboside. ROS; reactive oxygen species; WT; wildtype. SMC; smooth muscle cell.

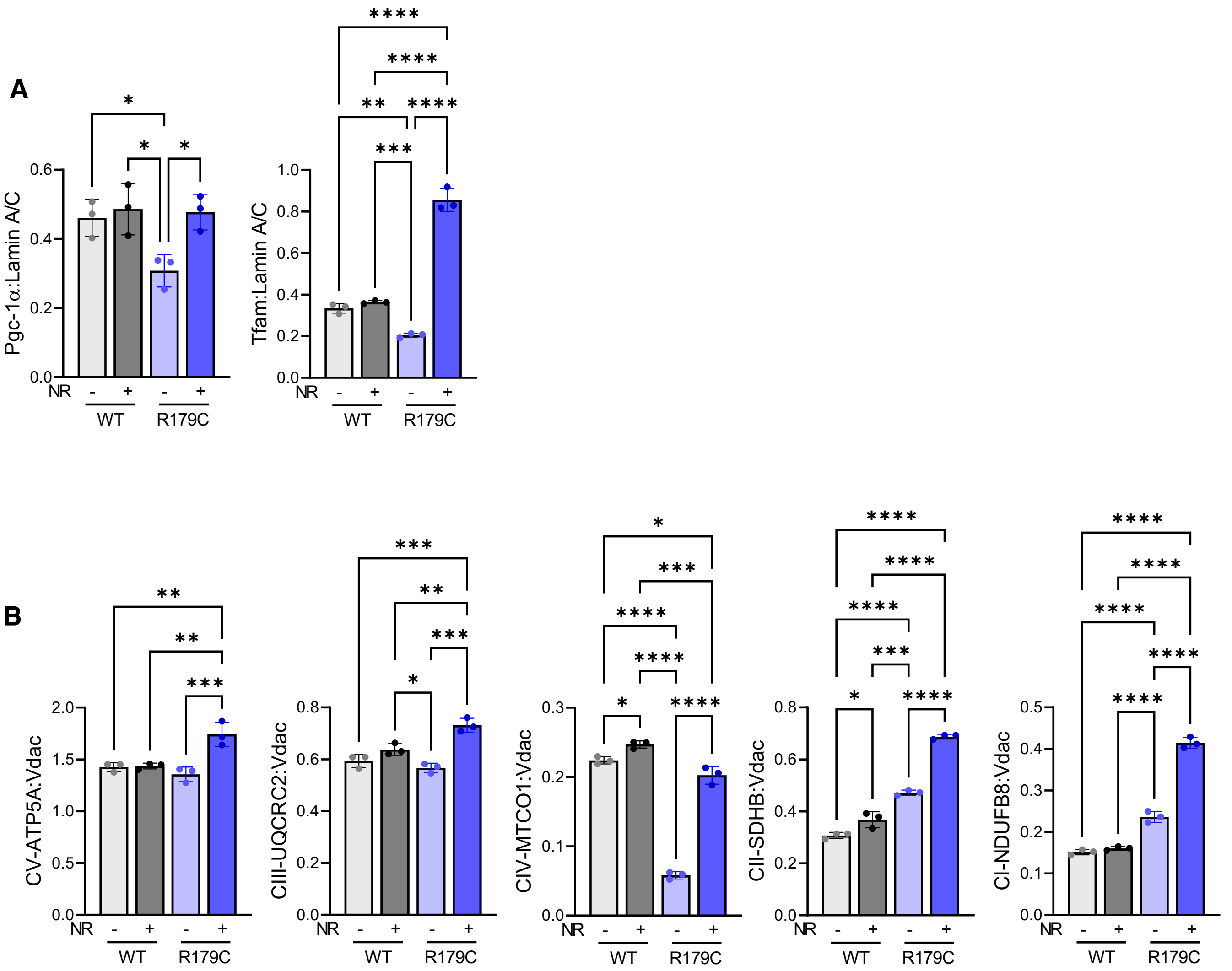

Supplemental 3. A. Western blot band quantification for Fig. 3A. **B.** Western blot band quantification for Fig. 3I.

932

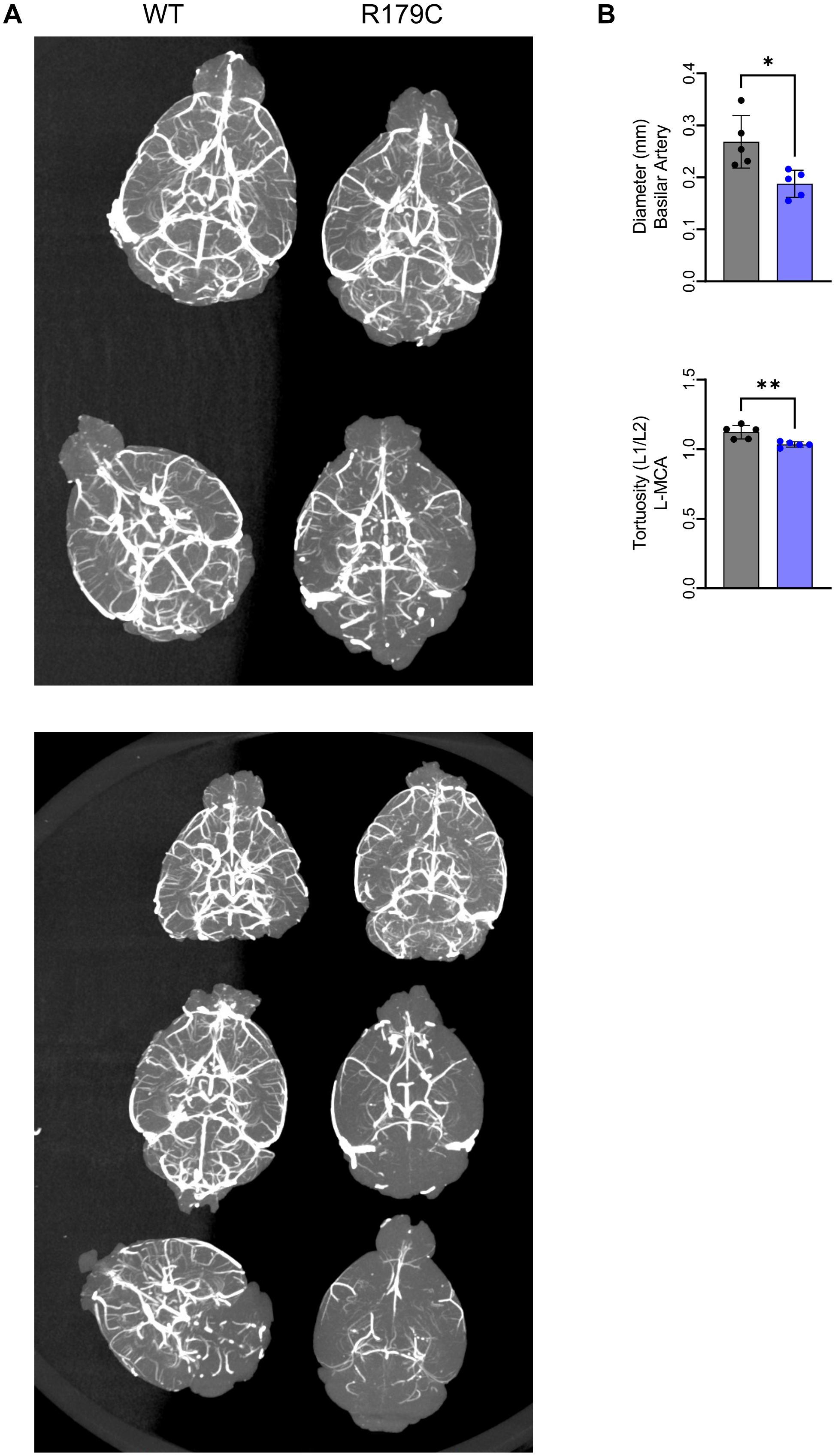

Supplemental 4. A. µ-CT images of Microfil-perfused 8-week old WT and *Acta2*$^{SMC\text{-}R179C/+}$ mice at baseline. **B.** *Acta2*$^{SMC\text{-}R179C/+}$ mice have reduced basilar artery diameter and L-MCA tortuosity compared to WT mice. µ-CT; micro computed tomography. WT; wildtype. L-MCA; left middle cerebral artery.

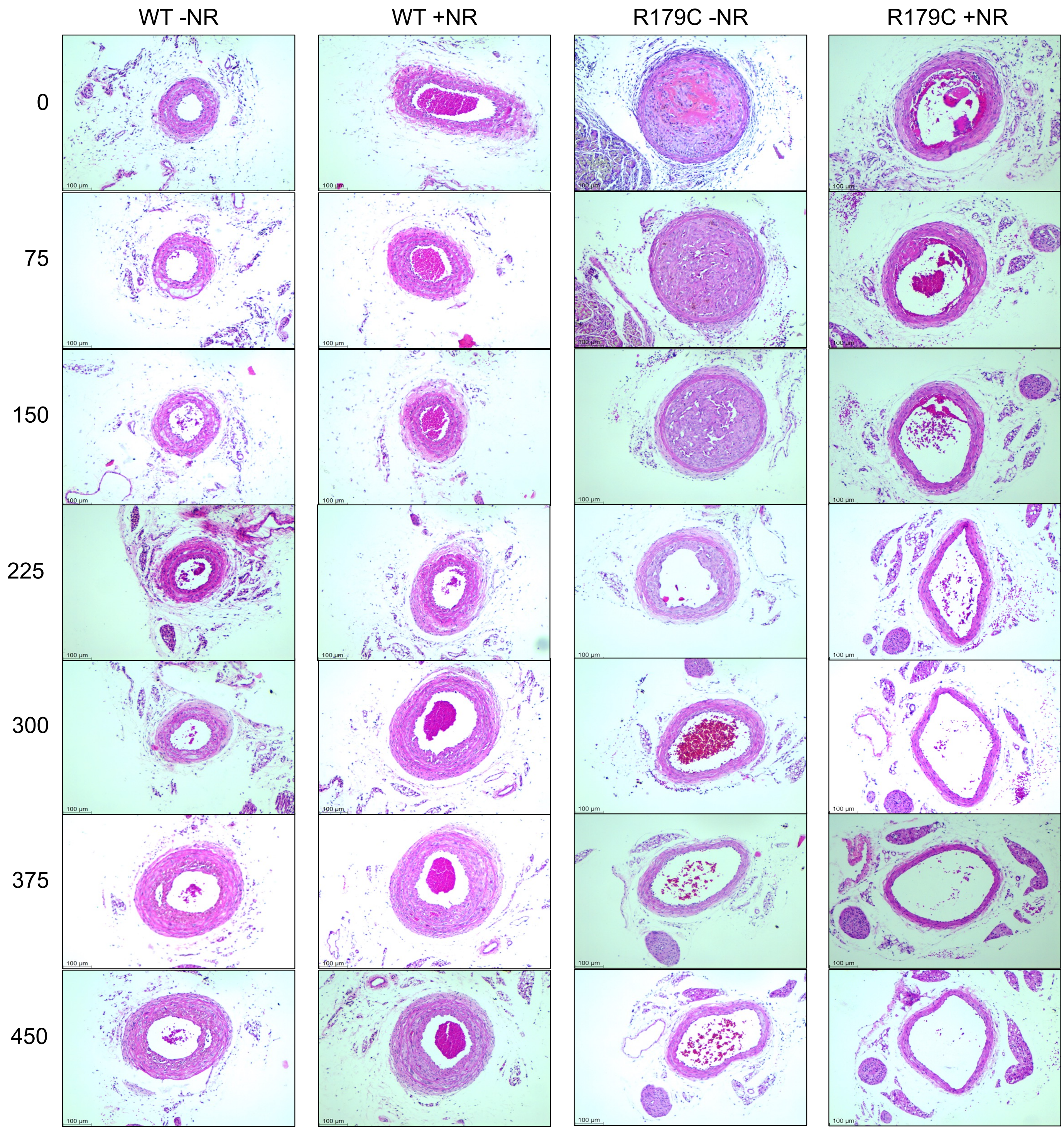

934

Supplemental 5. Representative images of left carotid arteries sectioned along the axial length. *Acta2*$^{SMC\text{-}R179C/+}$ mice exhibit intraluminal neointima-thrombus lesions, medial thinning, and increased lumen area proximal to ligation site 21 days post-LCAL, compared to WT mice which exhibit medial hypertrophy and patent lumens. NR reduces intraluminal lesions observed in *Acta2*$^{SMC\text{-}R179C/+}$ mice 21 days post-LCAL without affecting medial thickness and lumen area proximal to the ligation site. LCAL; left carotid artery ligation. WT; wildtype. NR; nicotinamide riboside.

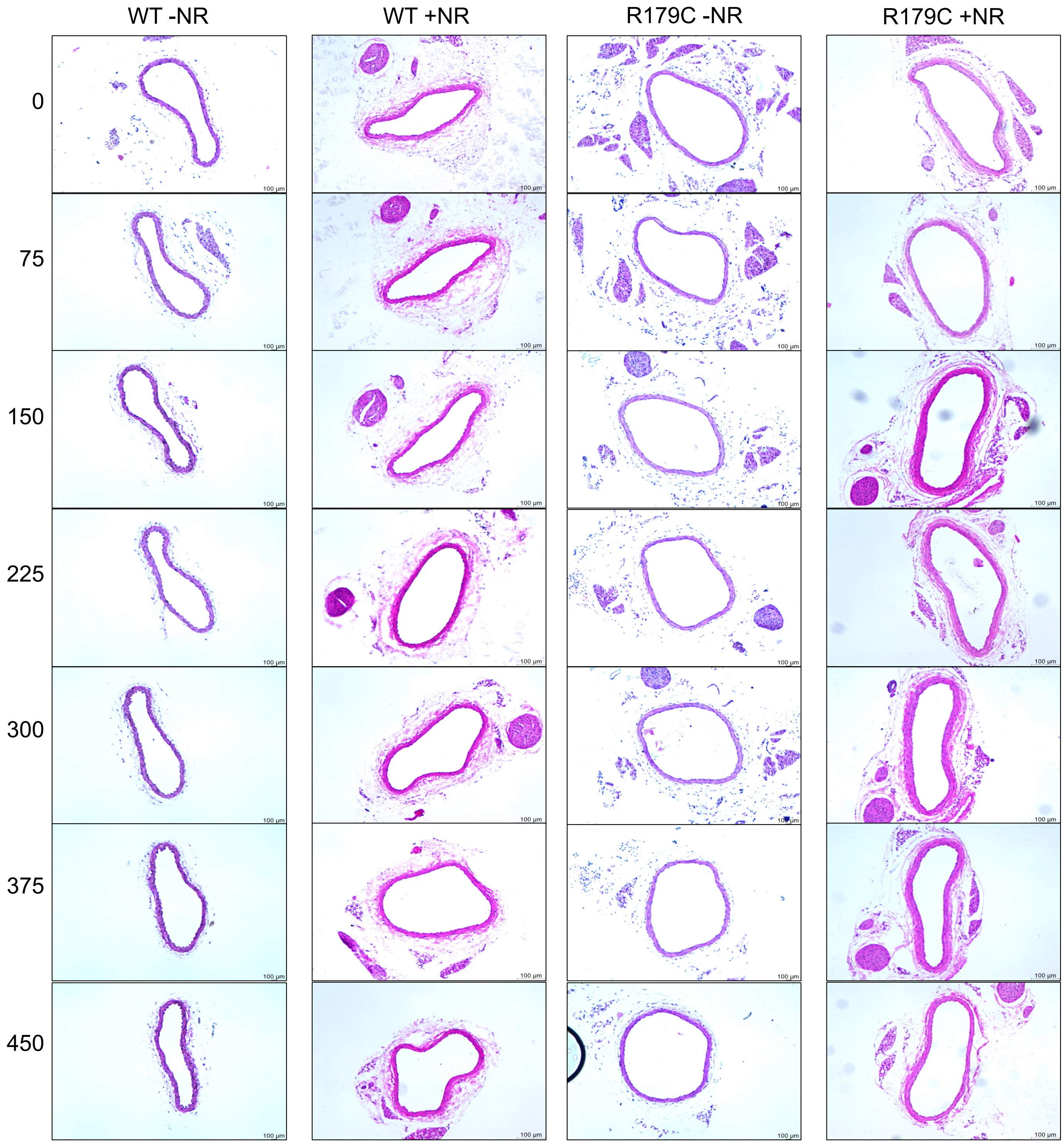

Supplemental 6. Representative images of right carotid arteries sectioned along the axial length of vehicle- and NR-treated LCAL-injured WT and *Acta2*$^{SMC\text{-}R179C/+}$ mice. NR; nicotinamide riboside. LCAL; left carotid artery ligation. WT; wildtype.

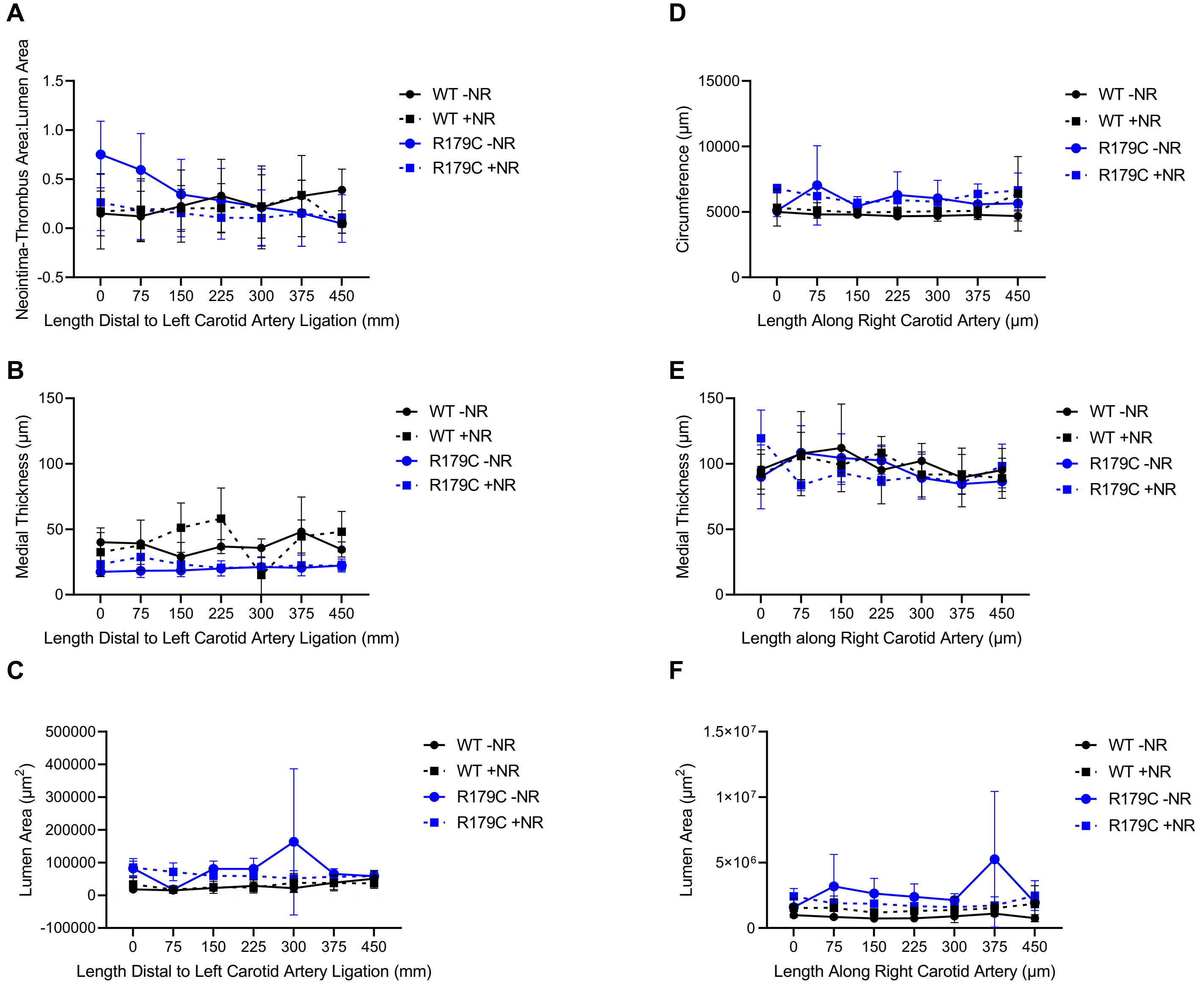

Supplemental 7. Left and right carotid arteries from vehicle- and NR-treated LCAL-injured WT and *Acta2*$^{SMC\text{-}R179C/+}$were serial sectioned transversely from the ligation site to the proximal end of the artery along the entire axial length and sampled images every 75 µm for hematoxylin and eosin staining starting at the ligation site. Occlusion percent, medial thickness, and lumen area were assessed along the length of the left (**A-C**) and right (**D-F**) carotid arteries. NR; nicotinamide riboside. LCAL; left carotid artery ligation. WT; wildtype.

A

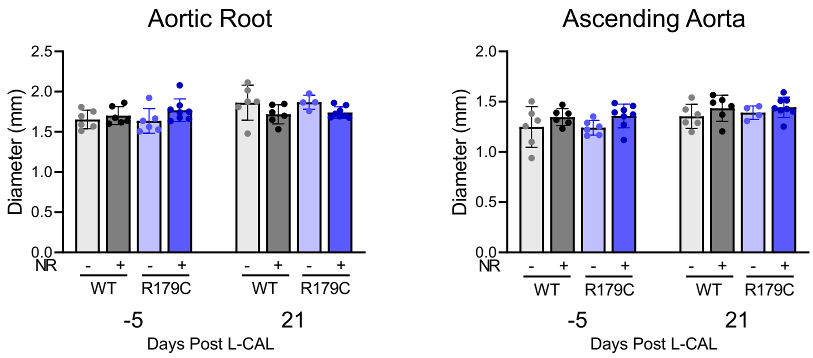

B

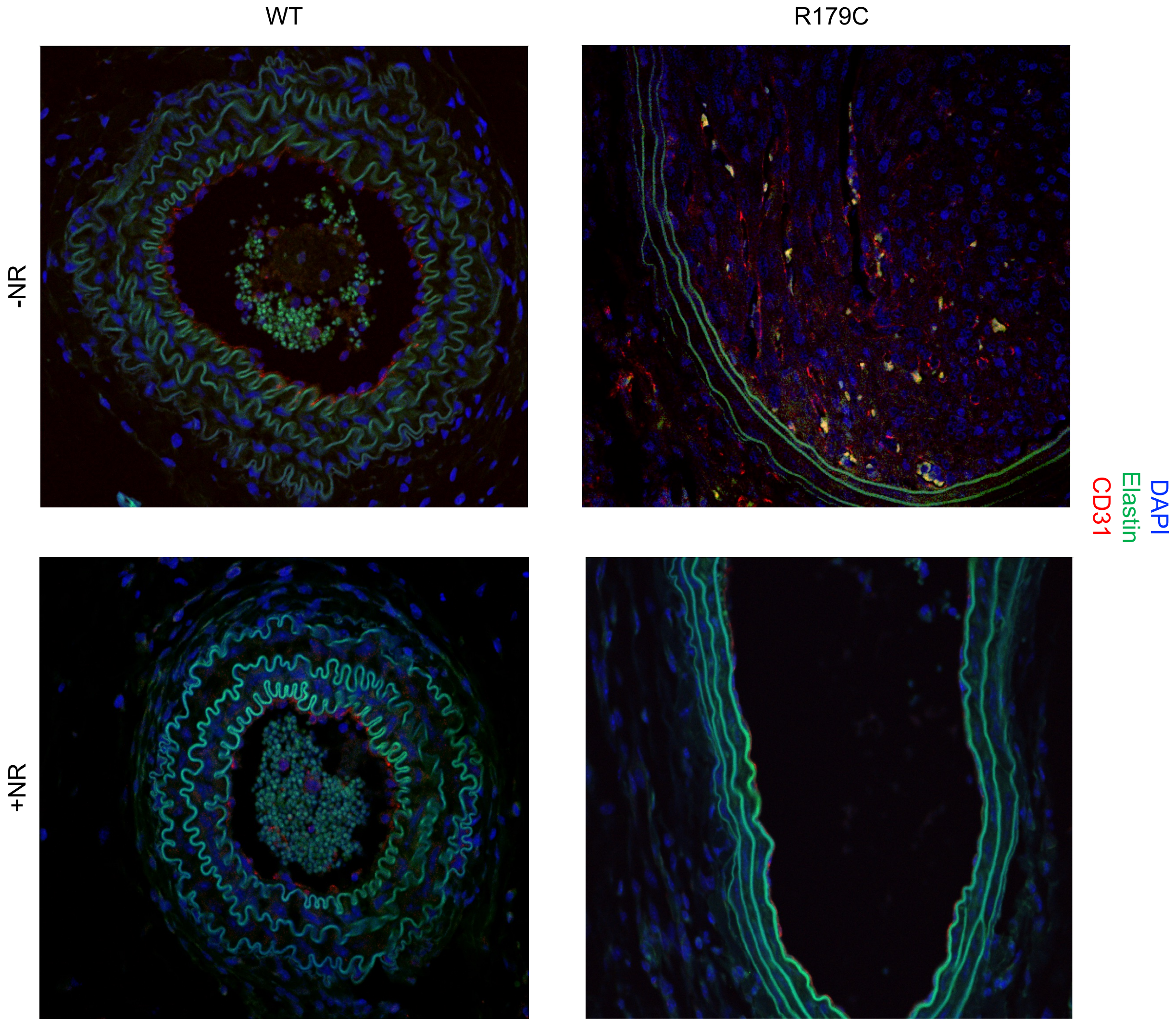

Supplemental 8. A. NR does not affect aortic root or ascending aortic diameter over a 26-day treatment period in LCAL-injured WT or *Acta2*$^{SMC\text{-}R179C/+}$ mice. **B.** 40X imaging of CD31-stained left carotid arteries. NR; nicotinamide riboside. LCAL; left carotid artery ligation. WT; wildtype.

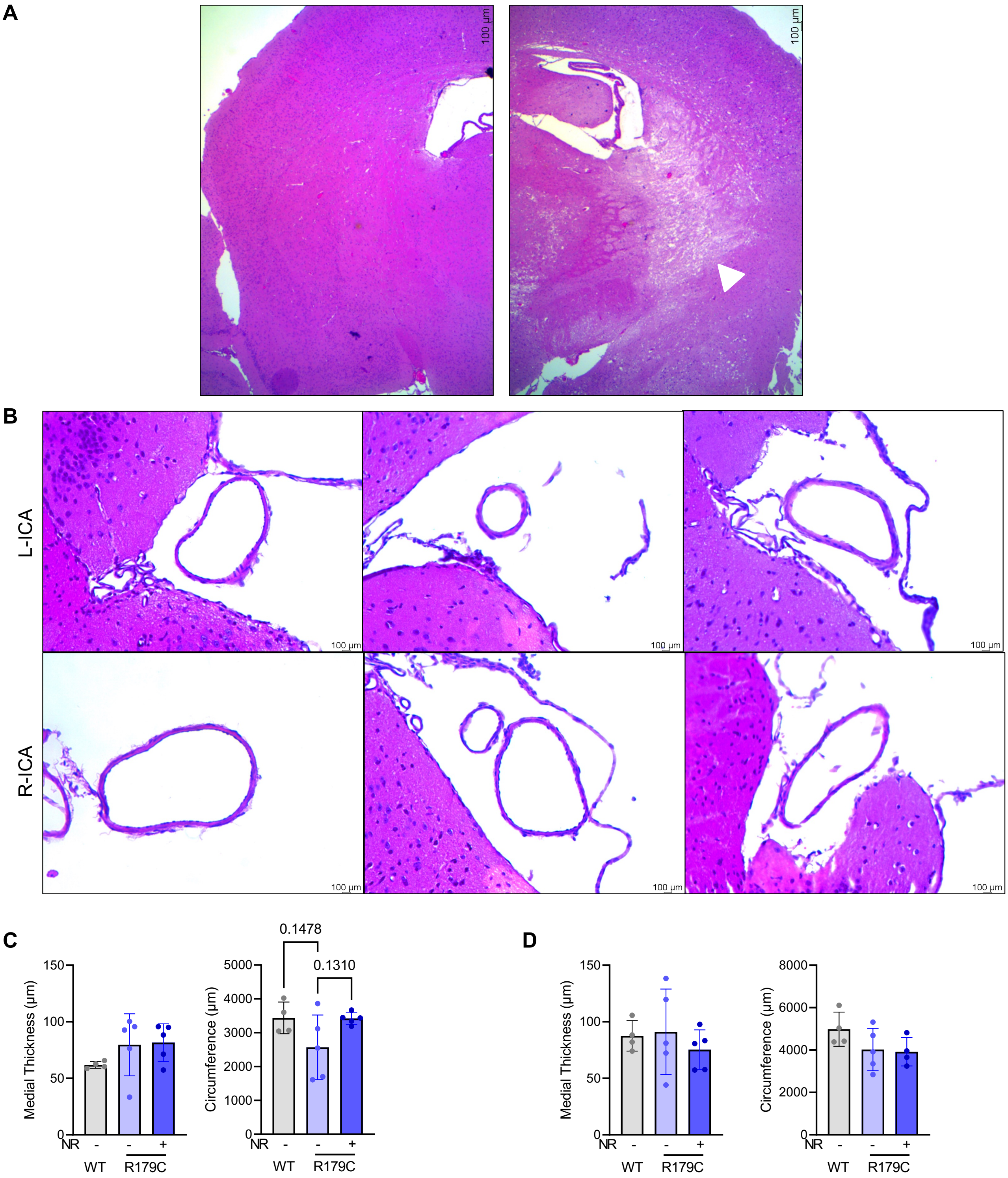

Supplemental 9. A. Hematoxylin and eosin staining of coronal section of brain from untreated *Acta2*$^{SMC\text{-}R179C/+}$ mice that died 4 days post-LCAL shows large, necrotic area of infarct (white arrowhead) in left hemisphere post-LCAL (right), compared to a normal-appearing right hemisphere (left), consistent with ischemic stroke due to LCAL. **B.** Hematoxylin and eosin staining of left (top) and right (bottom) ICAs within the CoW. No significant differences exist in medial thickness between untreated WT and mutant left **(C)** and right (**D**) ICAs. Untreated *Acta2*$^{SMC\text{-}R179C/+}$ mice have a trend of reduced circumference of left ICA compared to WT ($p=0.14$) 21 days post-LCAL, which increases ($p=0.13$) with NR treatment (**C**), and there are no differences in right ICA circumference in all three groups (**D**). LCAL; left carotid artery ligation. ICA; internal carotid artery. CoW; Circle of Willis. WT; wildtype. NR; nicotinamide riboside. *$p<0.05$, **$p<0.01$, ***$p<0.001$, ****$p<0.0001$.

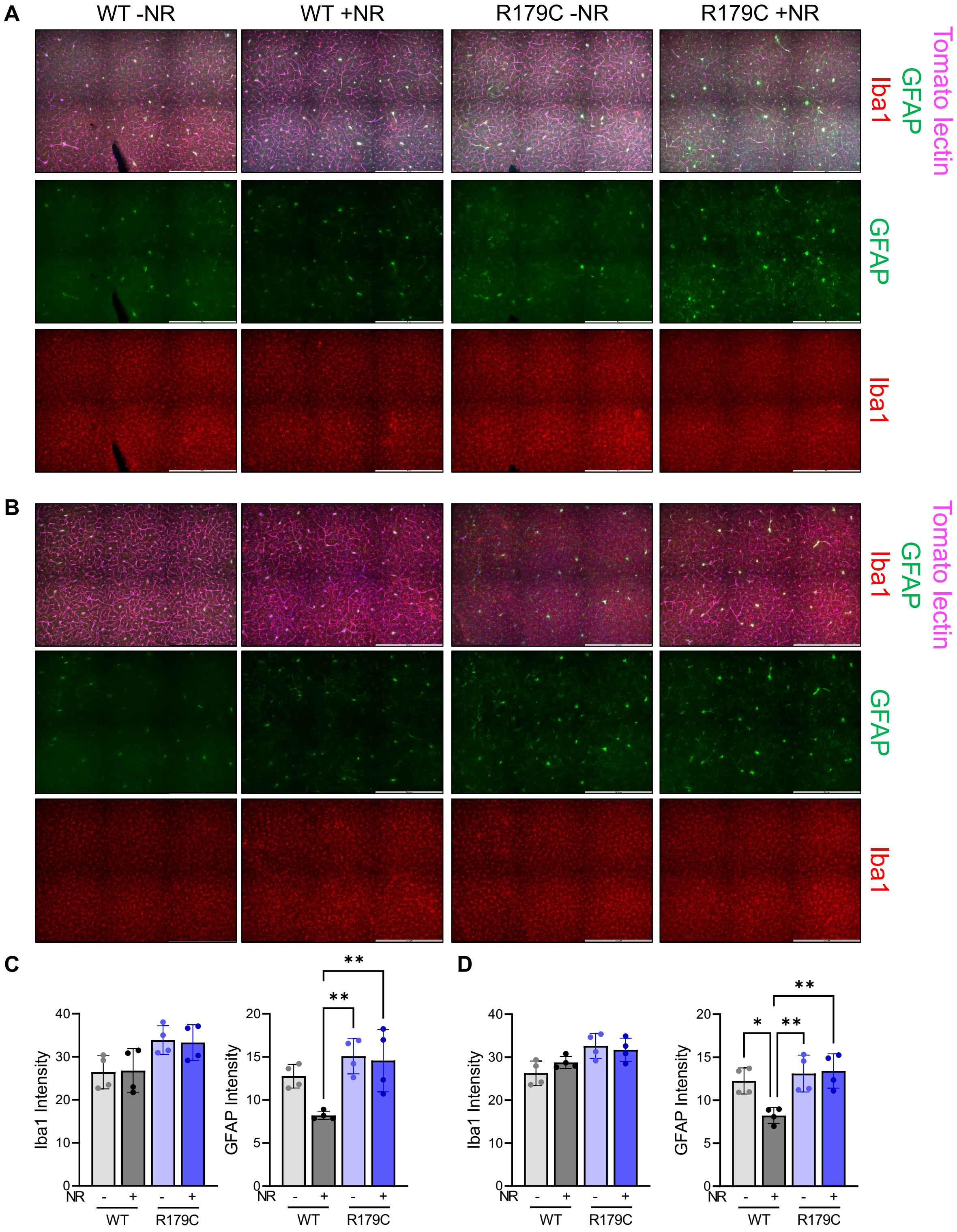

939 **Supplemental 10.** GFAP and Iba1 staining (**A**, left hemisphere; **B**, right hemisphere) quantification in vehicle- and NR-treated WT and *Acta2*$^{SMC\text{-}R179C/+}$ mice 21 days post-LCAL in the left (**C**) and right (**D**) cerebral hemispheres. WT; wildtype. NR; nicotinamide riboside. LCAL; left carotid artery ligation. *p<0.05, **p<0.01, ***p<0.001, ****p<0.0001.

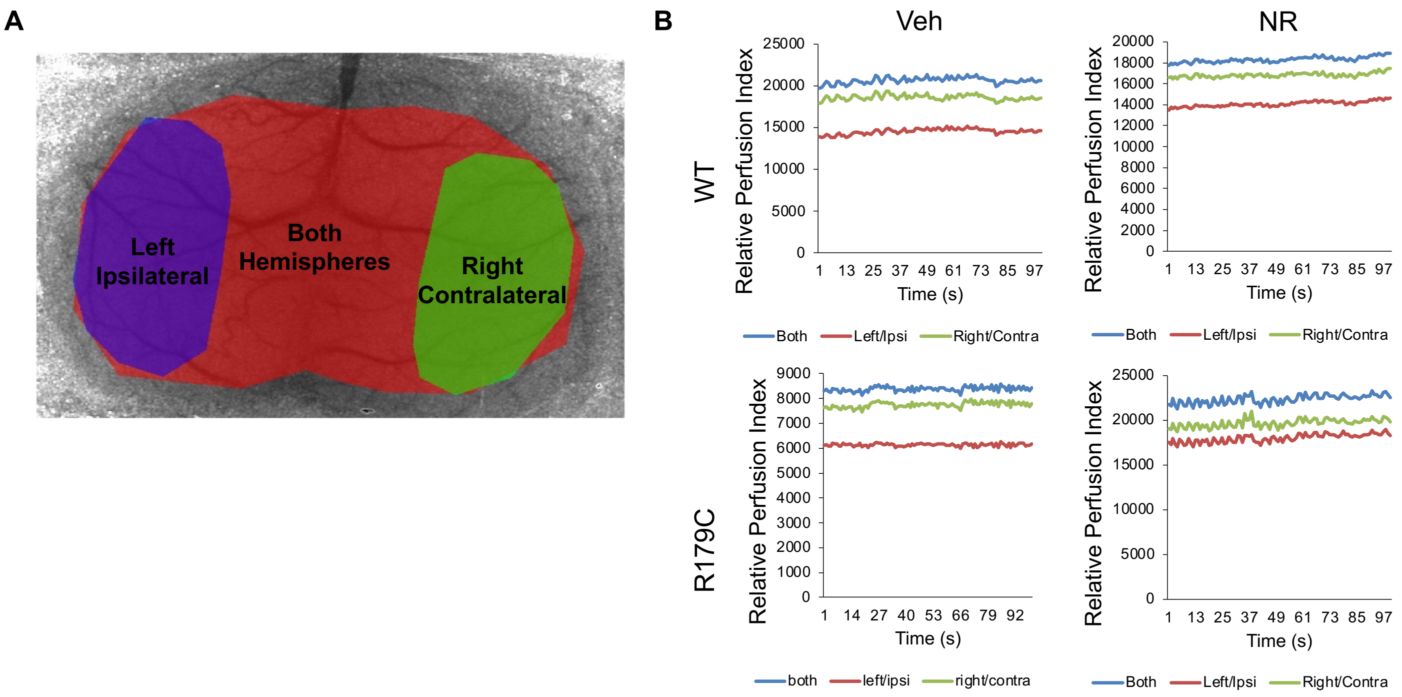

940

Supplemental 11. A. Regions of CBF measurement. **B.** Calculations of ipsilateral:contralateral CBF in vehicle- and NR-treated WT and *Acta2*$^{SMC\text{-}R179C/+}$ mice 21 days post LCAL. CBF; cerebral blood flow. NR; nicotinamide riboside. WT; wildtype. LCAL; left carotid artery ligation.

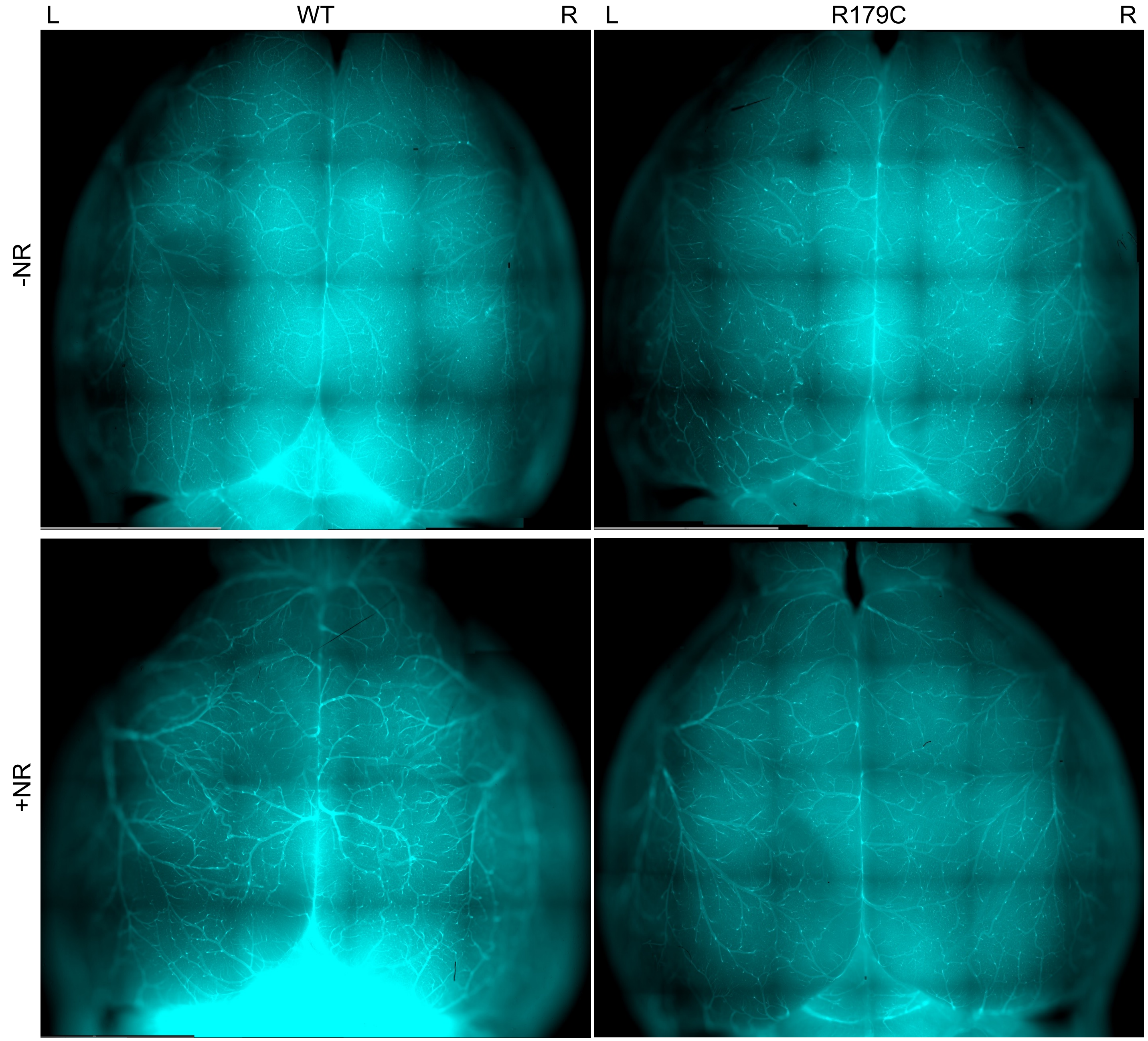

Supplemental 12. *Acta2*$^{SMC\text{-}R179C/+}$ mice increased left-sided leptomeningeal collateral remodeling 21 days post LCAL compared to WT. NR treatment reduces collateral remodeling in *Acta2*$^{SMC\text{-}R179C/+}$ mice that undergo LCAL. LCAL; left carotid artery ligation. WT; wildtype. NR; nicotinamide riboside.;
